# A Multiparadigm Approach to Characterize Dominance Behaviors in CD1 and C57BL6 Male Mice

**DOI:** 10.1523/ENEURO.0342-24.2024

**Published:** 2024-11-19

**Authors:** Meghan Cum, Jocelyn A. Santiago Pérez, Ryo L. Iwata, Naeliz Lopez, Aidan Higgs, Albert Li, Charles Ye, Erika Wangia, Elizabeth S. Wright, Catalina García Restrepo, Nancy Padilla-Coreano

**Affiliations:** ^1^ Department of Neuroscience, University of Florida, Gainesville, Florida 32610; ^2^ Department of Sociology, University of Chicago, Chicago, Illinois 60637

**Keywords:** dominance, mouse strain

## Abstract

Social status and dominance are critical factors influencing well-being and survival across multiple species. However, dominance behaviors vary widely across species, from elaborate feather displays in birds to aggression in chimps. To effectively study dominance, it is essential to clearly define and reliably measure dominance behaviors. In laboratory settings, C57BL/6 mice are commonly used to study dominance due to their stable and linear social hierarchies. However, other mouse strains are also used for laboratory research. Despite substantial evidence for strain effects on behavioral repertoires, the impact of strain on dominance in mice remains largely unstudied. To address this gap, we compared dominance behaviors between CD1 and C57BL/6 male mice across four assays: observation of agonistic behaviors, urine marking, tube test, and a reward competition. We found that CD1 mice demonstrate increased fighting, increased territorial marking through urination, and increased pushing and resisting in the tube test. We used unsupervised machine learning and pose estimation data from the reward competitions to uncover behavioral differences across strains and across rank differences between competing pairs. Of the four assays, urine marking and agonistic behaviors showed the strongest correlation with dominance in both strains. Most notably, we found that CD1 dominance rankings based on the tube test negatively correlated with rankings from all three other assays, suggesting that the tube test may measure a different behavior in CD1 mice. Our results highlight that behaviors can be strain-specific in mice and studies that measure social rank should consider assays carefully to promote reproducibility.

## Significance Statement

Recent studies have highlighted that social dominance can significantly impact behavior and the brain. As such, accurately measuring dominance behavior in laboratory settings is crucial in neuroscience research. In this study, we investigated the consistency of four dominance assays for male mice across two common mouse strains. We find that not all assays result in the same dominance rankings and dominance behaviors differ across strains. Our study sheds light on the importance of considering strains for assay selection, rigor, and reproducibility.

## Introduction

Across species, social status is associated with well-being and survival (Bartolomucci et al., 2001; Kozorovitskiy and Gould, 2004; Archie et al., 2012; Milewski et al., 2022). However, dominance behaviors manifest in different ways across species, from bird's elaborate feather displays (Woodcock et al., 2005; Loyau et al., 2007) to aggression in chimps (Muller and Wrangham, 2004). Some displays of dominance are conserved across species, such as linear hierarchies in chickens and mice (Rushen, 1982; Wang et al., 2011) or primary access to limited resources for dominant macaques and rats (Boccia et al., 1988; Costa et al., 2021). The last decade has seen an increase in neuroscience studies investigating social rank (Murlanova et al., 2022). Varying factors affect the mechanisms that govern dominance and social hierarchy, such as aggressive behavior and primary access to resources such as food, territory, and mates (Fulenwider et al., 2021).

Mice are commonly used to study dominance and rank as they form complex hierarchical and social structures in the wild (Hurst, 1987) and create stable hierarchies in laboratory settings (Wang et al., 2011; So et al., 2015). Social rank is associated with behavioral differences and health (W. Lee et al., 2022; Patel et al., 2024). Social rank also has sex-specific effects on stress responses where subordinate males and dominant females show greater resilience to chronic stress (Larrieu et al., 2017; Karamihalev et al., 2020). However, other studies have shown that subordinate mice show higher anxiety- and depression-related behaviors compared with dominant males (Yin et al., 2023), but a recent meta-analysis of the dominance literature indicates that there are no effects of rank on anxiety-like behavior, exploration, or learned helplessness behaviors (Varholick et al., 2021). This inconsistency across studies suggests that how dominance is measured may impact experimental findings and interpretation. Nonetheless, these studies demonstrate that social rank does modulate behavioral repertoire and how external cues are processed, highlighting that social dominance has a complex effect on social and nonsocial behavior.

Multiple dominance assays have prevailed in rodent laboratory research. Common assays to measure dominance in mice include observation of agonistic behaviors, the tube test (Lindzey et al., 1961; Wang et al., 2011), territory marking through urination (Desjardins et al., 1973; Hurst, 1990a), competition for resources (Wang et al., 2011; Li et al., 2022; Padilla-Coreano et al., 2022), ultrasonic vocalizations in the presence of females (Nyby et al., 1976), and the warm spot test (Zhou et al., 2017; LeClair et al., 2021; Battivelli et al., 2024), Many of these assays have been correlated and validated for the inbred C57BL/6 mouse strain (Wang et al., 2011; LeClair et al., 2021; Battivelli et al., 2024). However, there is ample literature indicating other behavioral differences across strains, including varying levels of sociability and social recognition (Netser et al., 2020; Liu et al., 2023; Mansk et al., 2023), which may impact social hierarchies. Levels of aggression vary widely across strains, with C57BL/6 mice exhibiting lower levels of intermale aggression compared with other inbred strains and outbred CD1 mice (Bisazza, 1981; Guillot and Chapouthier, 1996; Hsieh et al., 2017). Since mice show genetic variability within populations, it is important to consider how genetically distinct strains impact dominance behavior.

For this study, we focused on inbred male C57BL/6 (C57) mice and outbred male CD1 mice because they are commonly used in social neuroscience studies (Varholick et al., 2021; Cum et al., 2024) and form stable linear hierarchies (Wang et al., 2011; So et al., 2015; Williamson et al., 2016; Zhou et al., 2017; W. Lee et al., 2022). We selected four dominance-based assays to compare behavioral differences within and across strains: observation of agonistic behaviors, urine marking, tube test, and a trial-based reward competition assay (Padilla-Coreano et al., 2022). For the reward competition, we used SLEAP (Pereira et al., 2022), a machine learning tool used for pose estimation, to track behavior with higher granularity than human annotation. Unsupervised clustering on the competition data revealed differences across strains and ranks that were undetectable with traditional behavioral analysis, potentially highlighting a CD1-specific strategy for the reward competition task. Across the other three assays, CD1 mice showed greater dominance-related behaviors like pushing and territory marking compared with C57 mice. Most strikingly, we found a negative correlation between the results of the tube test and all three other assays for CD1 mice, suggesting that the tube test may not measure dominance within CD1 mice. Our results highlight the multifaceted nature of dominance and social rank as well as the importance of considering genetic background when selecting behavioral assays.

## Materials and Methods

### Animals

All experimental animals were housed in a 12 h reverse light cycle (8:30 A.M.–8:30 P.M.), and all behavioral experiments were conducted during the dark phase. All animals were male, 8 weeks old upon arrival to the laboratory, and group-housed in cages of three or four. Food and water were available *ad libitum* except during food restriction. CD1 (*n* = 63) mice were received from Charles River Laboratory, and C57BL/6 (*n* = 32) mice were received from The Jackson Laboratory. Mice were handled for at least 2 d prior to behavioral experiments. All behavioral experiments were conducted between 9:00 A.M. and 6:00 P.M. C57BL/6 mice were identified by unique bleach (Superior Preference) patterns, and CD1 mice were identified using unique patterns applied with a nontoxic animal marker (Stoelting Co.). Markings were reapplied as needed. All animal procedures were performed in accordance with the University of Florida animal care committee's regulations.

### Cohort overview

Three cohorts of mice were used in this study. Cohort 1 consisted of eight mice per strain. Cohorts 2 and 3 each had 12 mice per strain. For each cohort, behavioral experiments were run from 9 to 16 weeks of age (Table 1). All mice within a cohort were subjected to the same timeline, amount of training, and length of testing, unless otherwise specified. Urine marking and reward training/competition were never run on the same day to avoid lack of urination because of dehydration. A separate cohort of CD1 mice (*n* = 31) was used for the reward tube test experiment. This separate cohort was run in the rewarded tube test, agonistic behavior observations, and urine marking assay.

**Table 1.** Cohort ages

Cohort	Agonistic behaviors	Urine marking	Tube test	Reward competition
Cohort 1	12–15 weeks	14–16 weeks	9–12 weeks	12–14 weeks
Cohort 2	10–11 weeks	12 weeks	11–14 weeks	13–14 weeks
Cohort 3	11–14 weeks	12 weeks	10–14 weeks	13–15 weeks

Ages of mice during each behavioral assay across all three cohorts.

### Behavioral assays

#### Observation of agonistic behaviors

**Figure 1.**
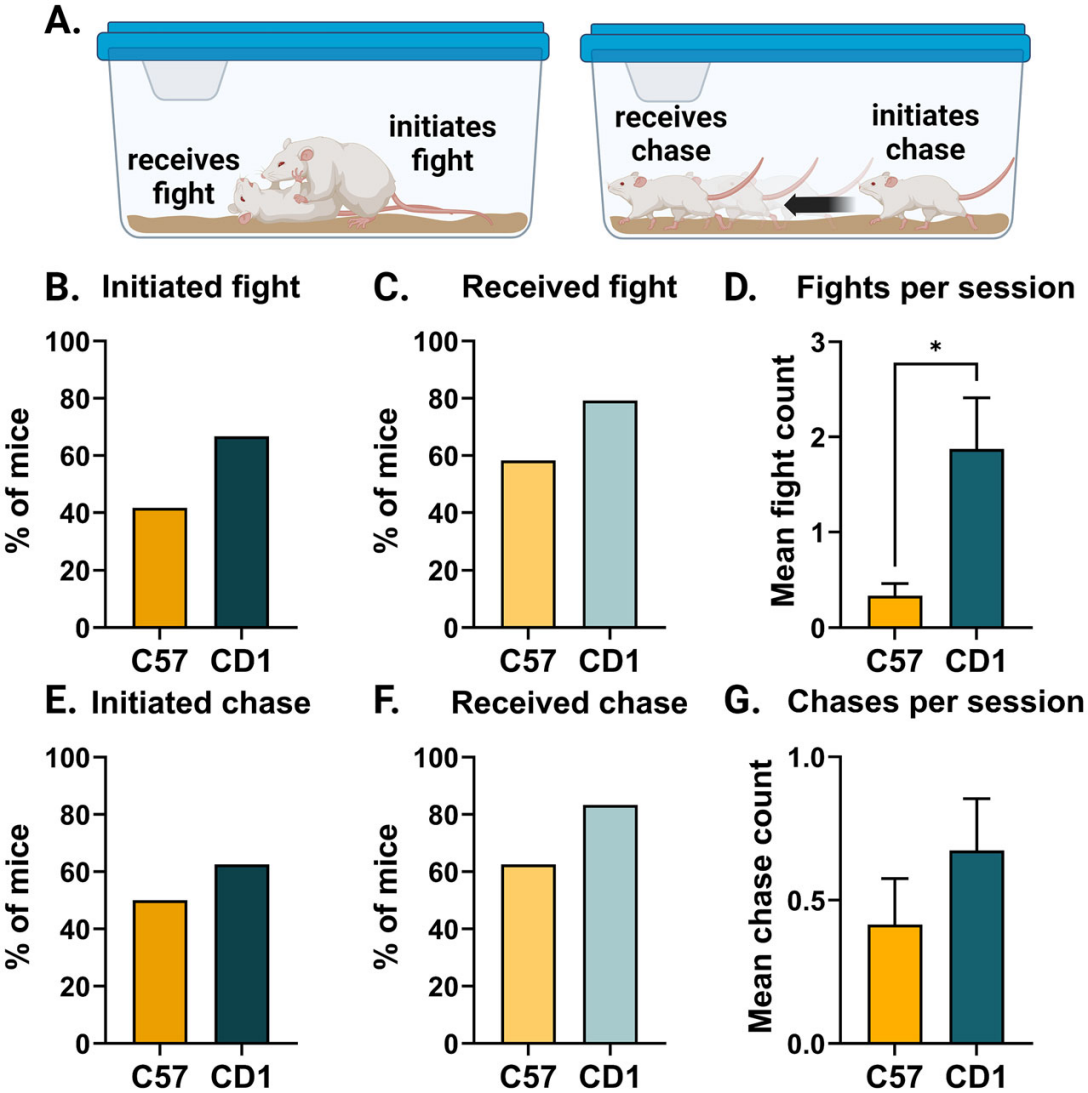
CD1 mice showed more agonistic behaviors in their homecage. ***A***, Schematic of agonistic behavioral events recorded: fighting (left) and chasing (right). ***B***, Percent of mice that initiated at least one fight during any observation (Fisher's exact test, *p* = 0.1468). ***C***, Percent of mice that received at least one fight during any observation (Fisher's exact test, *p* = 0.2124). ***D***, Average number of fights in a 30 min observation session per mouse (Mann–Whitney *U* = 175, *p* = 0.0137). ***E***, Percent of mice that initiated at least one chase across all observations (Fisher's exact test, *p* = 0.5612). ***F***, Percent of mice that received at least one chase across all observations (Fisher's exact test, *p* = 0.1930). ***G***, Average number of chases in a 30 min observation session (Mann–Whitney *U* = 226.5, *p* = 0.1885). For all plots, *n* = 24 C57 and *n* = 24 CD1. Plots show the mean, and error bars show the standard error of the mean. *p < 0.05, **p < 0.01, ***p < 0.001.

Mice were observed in a dimly lit and quiet room for 30 min. Interactions between cagemates were observed by trained observers. Fights and chases along with the initiator and receiver were recorded. All cages included in Figure 1 were observed for at least six sessions, and the mean fight and chase plots represent the average per mouse across all sessions. A subset of cages was subject to social opportunity manipulations. For the social opportunity condition, the mouse who had initiated the most fights prior to the social opportunity session was removed into a separate clean cage. If no fights had ever been initiated in a given cage, a mouse was chosen at random for removal. After 30 min of observation without the removed mouse, the mouse was returned to its homecage, and observations were recorded for an additional 15 min.

10.1523/ENEURO.0342-24.2024.f1-1 Figure 1-1 **Social opportunity’s effect on agonistic behaviors differs across strains. A.** Schematic of behavioral paradigm: 1) mouse who initiated the most fights was identified and placed into a separate cage. 2) observation of agonistic behaviors was recorded without the dominant mouse for 30 minutes. 3) The separated mouse was returned to the homecage, and agonistic behaviors were recorded for an additional 15 minutes. Data from observations during 2) removal of dominant and 3) return of the dominant were combined for 45 minutes of analyzed recordings. **B.** Percent of mice that initiated at least one fight during the observations (Fisher’s exact test, p = 0.0674). **C.** Percent of mice that received at least one fight during the observation (Fisher’s exact test, p = 0.1523). **D.** Number of fights in the 45-minute sessions per mouse (Mann-Whitney U = 114, p = 0.0521). **E.** Percent of mice that initiated at least one chase during the observations (Fisher’s exact; p = 0.2714). **F.** Percent of mice that received at least one chase during the observation (Fisher’s exact; p = 0.4774). **G.** Number of chases during the 45-minute session (Mann-Whitney U = 128.5, p = 0.1795). Plots show mean and error bars show standard error of the mean. For all plots, n = 12 C57 and n = 28 CD1. *p < 0.05, **p < 0.01, ***p < 0.001, # p < 0.1. Download Figure 1-1, TIF file.

#### Tube test

All training and testing were done in a dimly lit and quiet room. Mice were trained for 3–5 consecutive days prior to testing. Training consisted of walking through the tube, turning around at the other end, and returning through the tube such that the mouse walked the length of the tube eight times. A plastic cylinder, slightly smaller than the tube, was used to stop the mice from retreating or to encourage them to move forward by gently contacting their backs. Tube matches were conducted for at least 5 consecutive days and then sporadically to check stability and hierarchy maintenance. During tube testing, mice were subjected to one trial against each of their cagemates. The loser was defined as the first mouse to exit with all four feet from the tube. The order of the matches and starting side for each mouse was counterbalanced across days. Tube testing was run for 8–15 d (see Extended Data Fig. 3-1 showing all tube data). The tubes for C57 and CD1 mice were 30.48 cm long and made of clear polyvinyl chloride pipe. The inner diameter of the tube was 3.175 cm for C57 mice and 3.5 cm for CD1 mice. The tube was sanitized with 70% alcohol in between cages or, in the case of fecal or urinary matter, in between mice.

10.1523/ENEURO.0342-24.2024.f3-1 Figure 3-1 **Tube test stability across cohorts. A.** C57BL6 (yellow) cages across the three cohorts. Each colored line represents a mouse, and each point represents the total number of wins per day. **B.** CD1 (teal) cages across the three cohorts. Each colored line represents a mouse, and each point represents the total number of wins for that day. For all plots, there were four mice per cage and each mouse was subjected to three matches per day. Thin dashed lines separate the cohorts. Download Figure 3-1, TIF file.

In the rewarded tube test, 31 male CD1 mice, aged 8 weeks upon arrival, were used. All mice were food-restricted to 85% of their body weight, with 16 mice for the experimental (rewarded) group and 17 for the control (nonrewarded) group ran in two cohorts. Mice in the rewarded group were introduced to sugar pellets in their home cages before training to familiarize them with the new food. Individual training lasted 4–5 d with the sugar pellet or no pellet for controls, consistent within a cohort. During training trials, a sugar pellet was placed on the table at the exit of the tube so that the subject could retrieve it upon exiting the tube. Each day, training continued until each mouse ate eight pellets to ensure they associated reaching the end of the tube with receiving the pellet. For the first trial of the first day of training, the sugar pellet was placed right at the exit of the tube. For mice with longer latencies to traverse the tube, the pellet was placed at the tube's exit, while for mice that were crossing the tube quicker, the pellet was progressively farther away every trial, up to a maximum of 4 cm to reflect tube testing conditions. As performance improved, the distance was increased by 1 cm increments for each of the eight trials depending on the mouse's success in retrieving the previous pellet. For the control group, training followed the same procedure without providing the reward, and mice were paired for matches against their cagemates. Each mouse within a pair underwent identical tube training. Each cage was assigned either experimental or control, and matches were only against cagemates. In the rewarded tube test, once a mouse was pushed out of the tube, the loser was removed while simultaneously placing a sugar pellet 4 cm from the exit for the winner mouse.

#### Urine marking

Mice were in a dimly lit room in a 16.5 cm × 39.0 cm enclosure made of clear plastic with no floor. The enclosure was divided in half by a clear perforated wall such that the mice on either side of the perforated wall received visual and olfactory cues from the other. Chromatography paper (Whatman cellulose) was placed underneath the arena and underneath a wire mesh to prevent the mice from chewing the paper. Mice were left in the arena for 2–3 h. Urine marking session lengths were always consistent across strains and within cohorts. Urine markings were counted by hand under UV lights by trained counters. In the case that both mice had <20% difference or <5 urine spots difference, it was considered a tied match. Each pair within a cage was subjected to 1–2 trials. Each subject was run only once a day.

#### Reward competition

Mice were trained for 10 d prior to the reward competition. Mice were food-restricted to 85% of their body weight. Training sessions included individual mice being placed into a Med-PC operant chamber with an adapted 3D-printed reward port. Training sessions lasted 1 h. A tone of 10 s would play at 70 decibels at pseudorandomized intervals. A reward of 10 µl and 15 µl of vanilla Ensure for C57 and CD1 mice, respectively, was delivered 4 s after tone onset. Port entries were detected through infrared beam breaks. Latency to port entry and probability of port entry were calculated and assessed throughout training to confirm that the mice were learning to associate the tone with reward delivery. Importantly, there were no differences seen across strains in their ability to learn that the tone predicts the delivery of a reward, and all individual mice achieved an average latency of response of 10 s or less before competitions began. Mice were rotated across Med-PC operant chambers each day, and all cage members were trained at the same time. Competition sessions lasted 31 min (19–20 trials). Mice were subject to three competition sessions per day against each cagemate with at least a 2 h break in between sessions. The competition was run for 2 consecutive days. The order of matches and operant chambers was counterbalanced across days. Video of the training and competition was recorded at 30 fps.

### Data analysis

#### Tube test

Behavior was hand-scored by trained annotators using open-source Behavioral Observation Research Interactive Software (BORIS; Friard and Gamba, 2016). Two days of trials from Cohort 2 and Cohort 3 were included in the analysis, totaling 72 matches evenly split across 24 CD1 subjects and 24 C57 subjects. Each annotator scored 3–4 videos per strain. The trial start was defined as the moment when the experimenter releases both mice into the tube, and the trial end was defined as the moment when the loser mouse steps all four feet out of the tube. Push was defined as the mouse moving forward, attempting to move its feet forward, or darting its head forward at the opponent mouse. Resist was defined as the subject mouse not moving while being pushed by the opponent with no pushing behavior displayed by the subject. Retreat in contact was defined as moving backward while in contact with the opponent mouse. Passive retreat was defined as moving backward without contact with the opponent mouse.

#### Reward competition

Trained observers hand-scored each trial, assigning each trial a winner/loser or labeling the trial as a tie. A tie consisted of any trial in which neither mouse maintained a dominant position within the port (i.e., can reach the reward delivery spout) for longer than 1 s after the fourth second of the tone until tone offset. An uncontested trial was when one mouse was not at the port during the tone. SLEAP was used to annotate and predict body part positions during the reward competition for 16 CD1 sessions and 16 C57 sessions across 2 d of competition from Cohort 3. Six body parts were annotated: nose, forehead, left ear, right ear, thorax, and tail base. After SLEAP, missing locations for each node were interpolated and then smoothed using a Savitzky–Golay filter with a window of 25 frames using code on the SLEAP.ai website. For behavior state classification, seven features were extracted and normalized (*z*-score). The seven features were (1) the sum of the velocities of both mice based on the thorax; (2) the absolute value of the difference in velocity between the mice based on the thorax; (3) the sum of the distances between the port and nose node for each mouse; (4) the absolute value of the difference in distances between the port and nose node for each mouse; (5) the sum of the angle of orientation between the port, thorax, and nose node for each mouse; (6) the absolute value of the difference in the angle of orientation between the port, thorax, and nose node for each mouse; and (7) the distance between thoraxes between mice. The *z*-scored sum and absolute value of the differences were used as features to achieve agent invariance such that Mouse 1 being at the back wall and Mouse 2 being in the port versus Mouse 2 being in the port and Mouse 1 being at the back wall would provide similar values for Features 3 and 4. Additionally, the minimum angle with the thorax as midpoint was taken to achieve rotation invariance such that a 90° orientation clockwise from the port will have same the value as a 90° counterclockwise orientation from the port added to Features 5 and 6. Feature values were calculated for every third frame across the 32 videos. *K*-means clustering was applied to the seven feature values. *K*-means clustering was used as it clustered all data points and allowed us to control how many clusters or behavioral states it would produce. Trained observers watched videos constructed of 600 randomly selected frames from each cluster (Extended Data Movies 1–8). The number of clusters was decided based on the interpretability of the resulting videos, using the maximum number of clusters that resulted in videos that could be uniquely described by the trained observers. Uniform Manifold Approximation and Projection (UMAP) dimensionality reduction was applied for visualization after clustering. UMAP.fit_transfom() from the Python package umap-learn, version 0.5.5, was used. Default parameters were used except for random_state, which was defined as 42 to avoid stochasticity during plotting. Importantly, the dimensionality reduction was just for visualization, and the clustering was done using all seven features; therefore, the dimensionality reduction has no effects on cluster assignments. For rank differences, Elo scores were calculated for total rewards won across the reward competition and then simplified into a 1–4 rank; there were no tied ranks. For the percent enriched calculations, the formula used was as follows: (frames for rank difference *i* in cluster *j* / total frames for rank difference *i*) − (total frames in cluster *j* / total frames).

**Movie 1.** Randomly selected representative frames from cluster 0.

**Movie 2.** Randomly selected representative frames from cluster 1.

**Movie 3.** Randomly selected representative frames from cluster 2.

**Movie 4.** Randomly selected representative frames from cluster 3.

**Movie 5.** Randomly selected representative frames from cluster 4.

**Movie 6.** Randomly selected representative frames from cluster 5.

**Movie 7.** Randomly selected representative frames from cluster 6.

**Movie 8.** Randomly selected representative frames from cluster 7.

#### Directional consistency index

The directional consistency index (DCI) was calculated using the R package compete v0.1 (Curley, 2016).

#### Stability

The stability criteria for a given dyad for each of the four assays were as follows: agonistic behavior, having at least three interactions and 75% of fights or chases initiated by the same mouse; urine marking, having consistent and nontied outcomes across all trials; tube test, 75% of matches resulted in the same winner; and reward competition, 60% of trials across matches during Day 1 and Day 2 resulted in the same winner, excluding tied trials.

#### Elo score and David score

The Elo score and David score were calculated using custom Python scripts. We chose to present the Elo ranking system as it can account for ties and temporal shifts and has a baseline Elo score for cages that did not fight (1,000). This was important as the reward competition had many ties that would otherwise not be counted toward rank as well as three cages that did not fight during agonistic behavioral observations that also would not have been included in any correlation analyses. For agonistic behaviors, all observations were included in Elo score calculations including social opportunity manipulation observations.

Elo score equation:

$$E_{A}=\;\frac{1}{1+10^{\frac{R_{A}-R_{B}}{400}}},$$

$$R_{A}^{\prime }=\;R_{A}+K(S_{A}-\;E_{A}),$$

where the subscripts A and B represent the two opponents, *E*_A_ is the expected probability that A will win. *R*_A_ and *R*_B_ are the current ratings of the two opponents. *R*′_A_ will be the updated rating of A given the outcome. *S*_A_ will equal 1 is A wins, 0 if A loses, and 0.5 if the match is a tie. 400 and *K* are constants that control the sensitivity of rating changes; we used *K* = 20. All mice were assigned an Elo score of 1,000 to start.

David score equation:

$$DS_{i}=w_{i}+w_{i2}-l_{i}-l_{i2},$$

$$P_{ij}=\;\frac{\alpha_{ij}}{\mathrm{total}\;\mathrm{wins}\;\mathrm{for}\;i},$$

$$w_{i}=\;\overset{n}{\underset{\;j=1}{\sum }}\;P_{ij},$$

$$w_{i2}=\;\overset{n}{\underset{\;j=1}{\sum }}\;P_{ij}w_{i},$$

$$l_{i}=\;\overset{n}{\underset{\;j=1}{\sum }}\;1-P_{ij},$$

$$l_{i2}=\;\overset{n}{\underset{\;j=1}{\sum }}\;(1-P_{ij})w_{i},$$

Assuming you have *n* subjects, α is a matrix with shape *n* by *n*. Values in α represent total wins between the subjects, such that the value at row *i* and column *j*, *α_ij_*, is the number of wins an individual *i* has against individual *j*. Conversely, *α_ji_*, the value at row *j* and column *i*, is the number of wins *j* has against *i*. This is used to construct a matrix, *P*, of probabilities such that *P_ij_* is the probability of *i* winning against *j*. DS*_i_* represents the David score for individual *i*.

### Statistical analysis

#### Agonistic behaviors

A two-tailed Fisher's exact test was applied to all initiated/received fight and chase proportions. Average fights and average chases per 30 min session were calculated per mouse (total fights OR chases / number of sessions) and then a two-tailed Mann–Whitney *U* test was applied to the averages. To facilitate the interpretation of data shown in Figure 1, these calculations did not include any data from social opportunity manipulations and included only sessions of 30 min of observation. For Extended Data Figure 1-1, data calculations were done on data from a single social opportunity manipulation which included 30 min of observation time without the separated mouse and 15 min of observations immediately after the separated mouse was returned to the homecage.

#### Urine marking

A mixed-effects linear model was fit to the urine spots data using the R package lme4. The number of observations was 292 from 64 subjects. The model predicted the number of urine spots with fixed effects of strain, outcome (win/loss), and the interaction between the two, with a random effect of subject (repeated measure). Models with random effects of opponent and cage were also tested. The model with only subject as a random effect had the lowest Akaike information criteria (AIC) score and therefore was chosen (Table 2).

**Table 2.** Urine marking mixed-effects linear model

Fixed effect	Mean squares	Degrees of freedom	*F* value	*p*-value
Outcome (win/loss)	66,916	287.91	32.1188	3.519 × 10^−8^
Strain	35,558	105.05	17.0674	7.264 × 10^−5^
Outcome and strain	9,606	287.91	4.6108	0.03261

Results from a Type III ANOVA using the Satterthwaite's method on a mixed-effects linear model predicting the number of urine spots with fixed effects of outcome (win or loss) and the strain of the subject and the outcome strain interaction. Random effects (repeated measures) of subject were also included in the model.

A *t* test was run between the estimated marginal means, and the resulting *p*-values were adjusted for multiple comparisons using the Holm–Bonferroni (for four tests) method using the R package emmeans, shown in the table below. Degrees of freedom were calculated using the Kenward–Roger method.

#### Tube test

A mixed-effects linear model was fit to the hand-scored BORIS behavioral data from the tube test using the R package lme4. For each behavior, the model with the lowest AIC score was chosen. For all four behaviors, the interaction between strain and outcome as the fixed effects was insignificant and thus dropped. For push, resist, and passive retreat, the linear model predicted the percent of the trial in which the behavior was observed with fixed effects of outcome and strain and random effects of subject and scorer. For retreat in contact, the scorer did not explain a significant amount of variance and thus was dropped from the model. For all behaviors, random effects of cage and day of recording were also tested, none of which explained significant variance for any behavior (Tables 3–6). A *t* test was run using between the estimated marginal means, and the resulting *p*-values were adjusted using the Holm–Bonferroni method using the emmeans R package. Degrees of freedom were calculated using the Kenward–Roger method.

**Table 3.** Push mixed-effects linear model

Fixed effect	Mean squares	Degrees of freedom	*F* value	*p*-value
Outcome (win/loss)	0.31375	265.579	12.5551	0.0004667
Strain	0.16800	44.156	6.7631	0.0126129

Number of observations 288, with 48 subjects and 10 scorers. Results from a Type III ANOVA using the Satterthwaite's method on a mixed-effects linear model predicting the percentage of a trial the subject was pushing with fixed effects of outcome (win or loss) and the strain of the subject. Random effects (repeated measures) of subject and scorer were also included in the model.

**Table 4.** Resist mixed-effects linear model

Fixed effect	Mean squares	Degrees of freedom	*F* value	*p*-value
Outcome (win/loss)	0.010076	264.124	0.6761	0.4117
Strain	0.105588	41.608	7.0851	0.0110

Resist: number of observations 288, with 48 subjects and 10 scorers. Results from a Type III ANOVA using the Satterthwaite's method on a mixed-effects linear model predicting the percentage of a trial the subject was resisting being pushed with fixed effects of outcome (win or loss) and the strain of the subject. Random effects (repeated measures) of subject and scorer were also included in the model.

**Table 5.** Retreat in contact mixed-effects linear model

Fixed effect	Mean squares	Degrees of freedom	*F* value	*p*-value
Outcome (win/loss)	0.51076	263.12	60.423	1.729 × 10^−13^
Strain	0.00283	45.17	0.335	0.5656

Retreat (in contact): number of observations 288, with 48 subjects. Results from a Type III ANOVA using the Satterthwaite's method on a mixed-effects linear model predicting the percentage of a trial the subject was retreating in contact with fixed effects of outcome (win or loss) and the strain of the subject. Random effects (repeated measures) of subject were also included in the model.

**Table 6.** Push mixed-effects linear model

Fixed effect	Mean squares	Degrees of freedom	*F* value	*p*-value
Outcome (win/loss)	0.43913	262.084	44.2564	1.672 × 10^−10^
Strain	0.02103	43.414	2.1198	0.1526

Passive retreat: number of observations 288, with 48 subjects and 10 scorers. Results from a Type III ANOVA using the Satterthwaite's method on a mixed-effects linear model predicting the percentage of a trial the subject was passively retreating with fixed effects of outcome (win or loss) and the strain of the subject. Random effects (repeated measures) of subject and scorer were also included in the model.

#### Reward competition

Trials were hand-annotated as contested, tied, or uncontested by trained observers for the first day of the competition. A chi-square test was done on a 2 × 3 contingency table. For the percentage of rewards won, ties were excluded. The mouse that won over 50% of the trials was labeled as the winner, and the other the loser. If two mice won the same number of awards, one was assigned winner and the other loser. A mixed linear effects model was fit to predict the percentage of trials won with the outcome (winner/loser) and strain as fixed effects and subject as a random effect (repeated measures) using the R package lme4 (Table 7). Cage and opponent were also tested as random effects, but they did not contribute significantly to the model and were dropped. A *t* test was run using between the estimated marginal means, and the resulting *p*-values were adjusted using the Holm–Bonferroni method using the emmeans R package. Degrees of freedom were calculated using the Kenward–Roger method.

**Table 7.** Reward competition mixed-effects linear model

Fixed effect	Mean squares	Degrees of freedom	*F* value	*p*-value
Outcome (win/loss)	17.14	366.35	818.0081	<2 × 10^−16^
Strain	0	60.42	0	1.000
Outcome and strain	0.0003	366.35	0.0159	0.8998

Results from a Type III ANOVA using the Satterthwaite's method on a mixed-effects linear model predicting the percentage of trials won by a subject with fixed effects of outcome (win or loss) and the strain of the subject. Random effects (repeated measures) of subject were also included in the model.

For average velocities, SLEAP pose estimation data were used to calculate velocity for each frame as described above. For each mouse, the average velocity across all recordings was measured and compared using an unpaired, two-tailed *t* test across 12 C57 mice and 12 CD1 mice. For comparing cluster distributions across recordings (*n* = 16 recordings per strain), a two-sided, unpaired *t* test was applied, and *p*-values were adjusted using the Bonferroni–Holm method. Cluster 4 was the only other cluster that had an adjusted *p*-value of <1 (*t*_(8)_ = 2.241, *p* = 0.228).

#### DCI

The directional consistency index for each assay was compared across strains using an unpaired, two-tailed *t* test, and *p*-values were adjusted using the Holm–Bonferroni method with four comparisons between C57 and CD1 DCIs.

#### Elo versus rank

A two-way ANOVA was applied to Elo scores across rank and strain. Rank was determined as simply the ranked order of Elo scores (i.e., the highest Elo score was denoted as Rank 1, and the lowest Elo score was denoted as Rank 4).

#### Stability

After stability criteria were applied to each pair, unstable versus stable pairs across strains were compared using a two-tailed Fisher's exact test. Across two assays, the proportion of pairs who maintained hierarchy relations versus those who reversed hierarchy relationships across strains was compared using a two-tailed Fisher's exact test. Pearson’s correlations were run to create dominance score correlation matrices.

## Results

### CD1 mice show increased agonistic behaviors

We first quantified dominance metrics based on agonistic behavioral observations from cages of four mice. For both strains, we observed interactions among cagemates in the homecage for at least six sessions of 30 min, noting who initiated and who received fights and chases (Fig. 1*A*). Although more CD1 mice initiated and received fights, no significant strain differences were observed (Fig. 1*B,C*). Consistent with previous reports (Hsieh et al., 2017; Razzoli et al., 2023), CD1 mice, on average, fought more per observation session than C57 mice (*U* = 175, *p* = 0.0137, Fig. 1*D*). No significant differences were observed in chasing behavior (Fig. 1*E–G*).

Previous research shows that across species (Maruska et al., 2013; Williamson et al., 2017), individuals quickly increase agonistic behavior during a social opportunity, i.e., when the top-ranking individual is not present. To quantify sensitivity to social opportunity in laboratory standard cages in C57 and CD1 mice, we removed the mouse that initiated the most fights from each cage and observed conspecific behavior both in their absence and after returning them to the cage (Extended Data Fig. 1-1*A*). During social opportunity manipulations, CD1 mice exhibited more fighting behavior per session as well as had a greater proportion of mice initiating fights compared with C57 mice. However, these differences were not significant, suggesting larger sample sizes are needed to observe these social opportunity differences in the lab setting (*p* = 0.052 and *p* = 0.067; Extended Data Fig. 1-1*B,D*). These data suggest that CD1 mice exhibit more agonistic behavior than C57 mice and suggest that C57 mice may be less responsive to social opportunities than CD1 mice.

### Dominant CD1 mice show more territorial markings than dominant C57 mice

**Figure 2.**
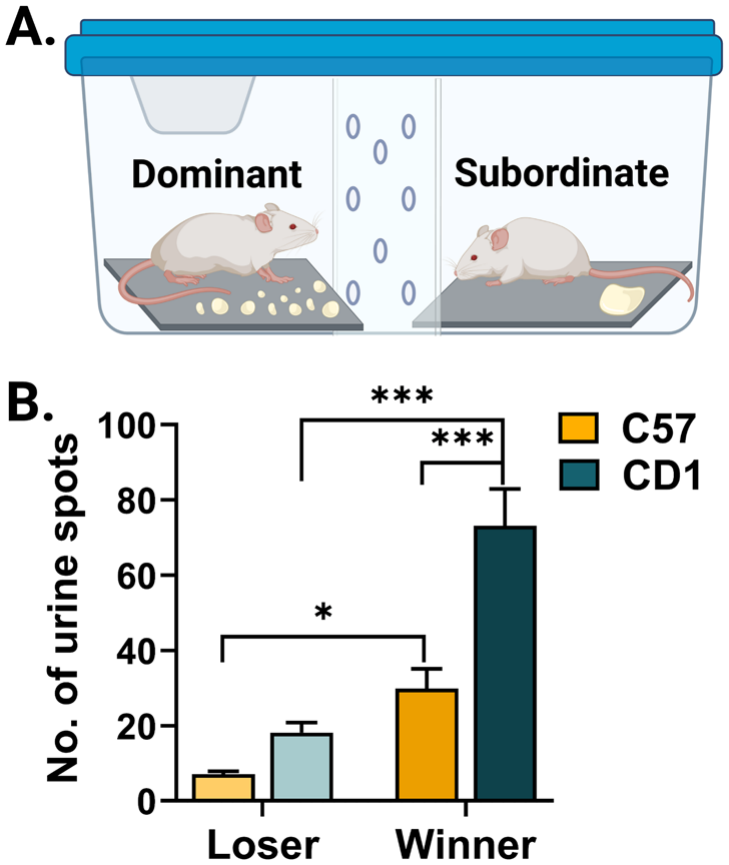
Territorial marking through urine marking is more prominent in CD1 mice. ***A***, Schematic of behavior paradigm showing two cagemates placed on either side of a perforated wall with the dominant mouse displaying the increased number of urine spots and the subordinate mouse displaying fewer urine spots. ***B***, The mean number of urine spots for each strain and outcome condition across 146 matches (*n* = 73 C57 matches from 32 mice, *n* = 73 CD1 matches from 32 mice). Each match had a winner and a loser based on the number of urine spots. Comparing estimated marginal means of a mixed-effects linear model, *p*-values adjusted for multiple comparisons using Holm–Bonferroni method: winner CD1 versus loser CD1, *t*_(287)_ = 5.486, adj. *p* < 0.0001; winner C57 versus loser C57, *t*_(288)_ = 2.471, adj. *p* = 0.0281; winner C57 versus winner CD1: *t*_(116)_ = −4.117, *p* = 0.0002; loser C57 versus loser CD1, *t*_(116)_ = −1.460, *p* = 0.1460. Plots show the mean, and error bars show the standard error of the mean. *p < 0.05, **p < 0.01, ***p < 0.001.

Many animals, including rodents, show territorial behavior such as territorial urine marking (Brashares and Arcese, 1999; Allen et al., 2016; Miaretsoa et al., 2022). Territorial marking through urination, both through the number of urine marks and the area of territory marked, has been previously associated with agonistic behaviors in male mice (Desjardins et al., 1973; Hurst, 1990a). Therefore, we next quantified dominance based on urine markings in C57 and CD1 mice. We placed two cagemates in a novel arena separated by a perforated wall (Fig. 2*A*) and quantified the number of urine markings made by each mouse (Desjardins et al., 1973; Wang et al., 2011). “Winner” CD1 mice—mice that produced more urine markings—had more total urine spots than “winner” C57 mice (*t*_(116)_ = −4.117, *p* = 0.0002; Fig. 2*B*). This difference was not observed in the “losers” across strains, suggesting that CD1 mice do not simply urinate more than the C57 mice indiscriminate of context but that either dominant CD1 mice exhibit more territorial marking due to the presence of subordinate mice or that subordinate CD1 mice decrease urination in the presence of dominant mice.

### CD1 mice push and resist more during the tube test

**Figure 3.**
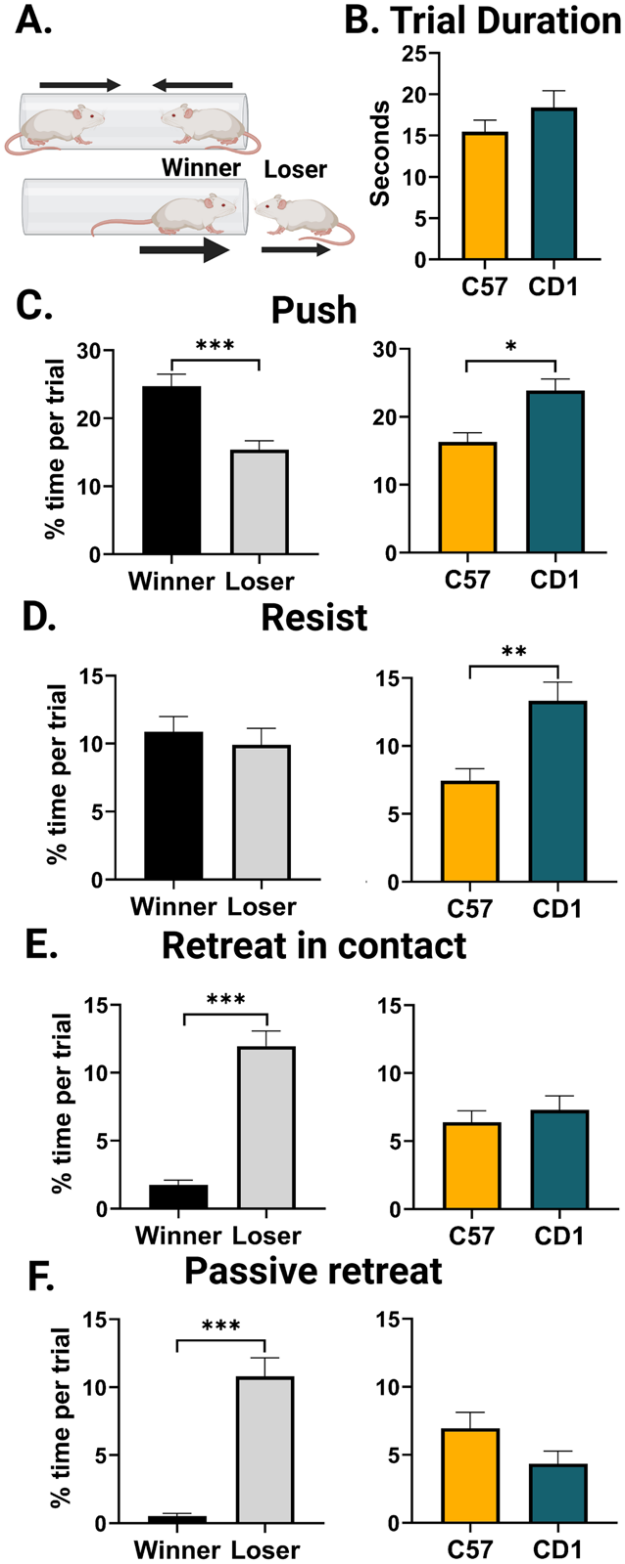
Tube test behaviors show minor strain differences. ***A***, Schematic of a tube test trial in which the loser is pushed out of the tube by the winner. ***B***, Mean trial duration for match length for C57 mice (mean, 15.46 s; SEM, 1.415 s) and CD1 mice (mean, 18.39 s; SEM, 2.033 s). ***C–E***, Average percentage of a trial spent in the given behavior is shown by the trial outcome and by strain. Winner (black) and loser (gray) averages were calculated from all data, while C57 (yellow) and CD1 (teal) averages were calculated by combining winners and losers. ***C***, Percent tube trial time spent pushing. Winner versus loser: *t*_(266)_ = −3.508, *p* = 0.0005. C57 versus CD1: *t*_(45)_ = −2.600, *p* = 0.0126. ***D***, Percent tube trial time spent resisting. Winner versus loser: *t*_(264)_ = 0.814, *p* = 0.463. C57 versus CD1: *t*_(44.9)_ = −2.662, *p* = 0.0107. ***E***, Percent tube trial time spent retreating in contact. Winners versus losers: *t*_(263)_ = 7.695, *p* < 0.0001. C57 versus CD1: *t*_(45.5)_ = −0.579, *p* = 0.5656. ***F***, Percent tube trial time spent passively retreating (without contact from a competitor). Winners versus losers: *t*_(263)_ = 1.456, *p* < 0.0001. C57 versus CD1: *t*_(44.8)_ = 1.456, *p* = 0.1524. Plots include data for *n* = 24 C57 mice and *n* = 24 CD1 mice across 144 trials; *n* = 72 C57 trials, and *n* = 72 CD1 trials. Plots show the mean, and error bars show the standard error of the mean. All *t* tests were calculated using estimated marginal means of a mixed-effects linear model, and reported *p*-values were adjusted for multiple comparisons using the Holm–Bonferroni method. *p < 0.05, **p < 0.01, ***p < 0.001.

Next, we analyzed dominance behavior during the tube test, a commonly used laboratory assay to measure social rank in mice (Lindzey et al., 1961; Wang et al., 2011; LeClair et al., 2021; Battivelli et al., 2024). After placing two mice into a small tube at either end, the mouse that proceeds forward and pushes the other mouse out the other end is the “winner.” Importantly, most past tube test reports are limited to C57 male mice. To assess potential strain differences, we compared tube test behavior between C57 and CD1 mice. We quantified four behaviors during the trials: push, resist, retreat in contact, and passive retreat. Trial duration did not differ across strains (*F*_(1,141)_ = 1.292, *p* = 0.2575; Fig. 3*B*), and there was no interaction between trial outcome (win/loss) and strain on time spent in any one behavior. However, two behaviors showed strain effects that were independent of trial outcome: CD1 mice spent more time during the trial pushing (*t*_(45)_ = −2.600, *p* = 0.0126) and resisting (*t*_(44.9)_ = −2.662, *p* = 0.0107) compared with C57 mice (Fig. 3*C,D*). As expected, winners across both strains pushed more than losers (*t*_(266)_ = −3.508, *p* = 0.0005), and losers retreated more than winners, both in contact (*t*_(263)_ = 7.695, *p* < 0.0001) and passively (*t*_(263)_ = 1.456, *p* < 0.0001; Fig. 3*C,E,F*). These data suggest that based on the typical behaviors quantified with the tube test there are minimal strain differences between C57 and CD1 mice.

### Unsupervised clustering reveals behavioral states with strain differences during the reward competition

**Figure 4.**
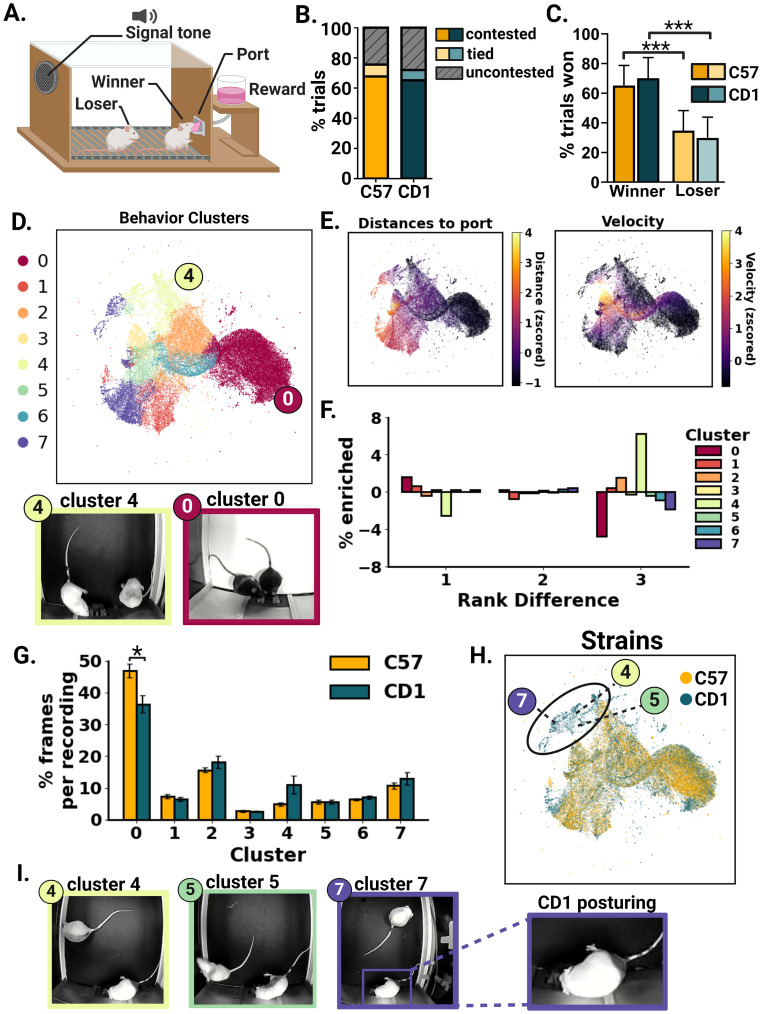
Reward competition behavioral states revealed with machine learning. ***A***, Schematic showing the reward competition where a signal tone indicates a reward to be delivered at the port. ***B***, Breakdown of the types of trials (contested, uncontested, and tied) across all matches from the first day of the competition with equal numbers of trials across strains [chi-square: *X*^2^(2, *N* = 1,824) = 3.46, *p* = 0.1773]. ***C***, The percentage of trials won across winners (won >50% of trials) and losers; *n* = 96 matches for C57 from 12 mice, and *n* = 96 matches for CD1 from 12 mice. C57 winners versus losers: paired *t* test, *t*_(378)_ = 20.292, *p* < 0.0001. CD1 winners versus losers: paired *t* test, *t*_(347)_ = 19.883, *p* < 0.0001. ***D***, UMAP plot where each data point is a frame across 32 recordings (16 matches per strain). Clusters were calculated on feature-based high-dimensional data using *K*-means clustering (see Materials and Methods). The colored circles indicate the clusters with example frames shown below: Cluster 4 (light green) and Cluster 0 (burgundy). ***E***, Features superimposed on the UMAP embedded space. Distances to the port are the *z*-scored sum of the distances to the port for both mice in each frame. Velocity is the sum of the *z*-scored speed for both mice in each frame. ***F***, The percentage of enrichment (see Materials and Methods) per cluster across rank differences is defined based on reward competition outcomes. ***G***, The average percentage of frames across all recordings (C57, *n* = 16; CD1, *n* = 16). Averages were calculated as (frames per cluster / total frames for each recording). Two-tailed unpaired, *t* tests were applied, *p*-values were adjusted using the Holm–Bonferroni method (Cluster 0: *t*_(8)_ = −3.048, adj. *p* = 0.0382). ***H***, Strain overlaid on the UMAP embedded space. The black circle indicates the area with heavy CD1 representation, with three clusters indicated by the colored circles. ***I***, Example frames from the circled region of the plot in ***H*** from indicated clusters. To the right is a zoomed-in version of an example Cluster 7 image illustrating the CD1 posture seen across all example images. In ***D***, ***E***, and ***H***, for visualization purposes, every 10th frame was plotted. In ***C***, ***F***, and ***G***, plots show means, and error bars show the standard error of the mean. *p < 0.05, **p < 0.01, ***p < 0.001.

An important aspect of social dominance is priority access to resources (Fulenwider et al., 2021). We recently developed a trial-based reward competition assay where mice compete for a reward signaled by a tone (Fig. 4*A*; Padilla-Coreano et al., 2022). However, this assay was previously only performed in C57 male mice. To assess potential strain differences, we compared C57 and CD1 behavior during the reward competition assay. Average weight loss by subjects did not correlate with dominance in either strain (Extended Data Fig. 4-1). Neither the percentage of trial types (contested, tied, or uncontested) nor the difference in total rewards obtained by the winner versus the loser differed by strain (trial type, *X*^2^ = 3.46, *p* = 0.1773, *N* = 1,824 trials; winner C57 vs winner CD1, *t*_(139)_ = 0.085, *p* = 1.00; loser C57 vs loser CD1, *t*_(139)_ = −0.085, *p* = 1.00; Fig. 4*B,C*). In addition, average velocities during the competition also did not differ between C57 and CD1 mice (*t*_(0.08934)_, *p* = 0.9296). To identify more nuanced behavioral differences that may occur during the reward competition, we used a machine learning approach. First, to track behavior with more granularity than human annotation, we used SLEAP (Pereira et al., 2022), a deep learning tool for pose estimation, to track the positions of both mice during the reward competition. This deeper quantitative analysis using machine learning showed behavioral differences not captured by binary outcomes such as win and loss, possibly uncovering a strain-specific strategy for navigating the reward competition. Based on the pose tracking, we calculated relevant features, such as velocity, distance to the reward port, and angles relative to the reward port (Extended Data Fig. 4-2*A,B*). *K*-means unsupervised clustering revealed behavioral states that were visualized using Uniform Manifold Approximation and Projection (UMAP) to reduce dimensionality (Fig. 4*D*). Behavioral states captured by this method included highly competitive states of both mice at the port captured by Cluster 0 (Fig. 4*D*), a lower competitive state where one mouse is in the port while the other is near but facing away from the port captured by Cluster 4 (Fig. 4*D*) and the lowest competitive state with the second mouse positioned along the back wall facing away from the reward port captured by Cluster 7 (Extended Data Fig. 4-2*C*). We overlaid the features on the UMAP space which more clearly shows the differences in the distance to the port and the velocity of the mice that define each behavioral state across clusters (Fig. 4*E*). To further confirm that each cluster showed distinct behavioral states, videos composed of frames from each cluster were observed for user interpretation and frames from each cluster are shown (Extended Data Movies 1–8; Extended Data Fig. 4-2*C*).

10.1523/ENEURO.0342-24.2024.f4-1 Figure 4-1 **Weight loss is not correlated with reward competition dominance. A.** Average weight loss in grams across reward competition days for C57 mice (n = 32) plotted against their final Elo score for reward competition (Pearson correlation r = 0.015, p = 0.9145). **B.** Average weight loss in grams across both competition days for CD1 mice (n = 32) plotted against their final Elo score for reward competition (Pearson correlation r = -0.114, p = 0.5338). Download Figure 4-1, TIF file.

10.1523/ENEURO.0342-24.2024.f4-2 Figure 4-2 **Reward competition SLEAP pipeline and unsupervised behavioral states. A.** SLEAP pipeline schematic from (1) using SLEAP for body part and ID tracking, (2) selecting and calculating features; 3 example features are shown (7 were used and are listed in the methods), (3) using K-Means clustering to create 8 clusters and (4) visualizing the output in UMAP embedding (4). **B.** Recording and feature overlays on the UMAP embedding data. Each data point is a video frame from n = 16 C57 match recordings and n = 16 CD1 match recordings. Top left, recordings plotted by color on the UMAP space. Top right and bottom: Features shown are: angle to port (sum) which is the sum of the angles to port across both mice, angle to port difference (diff) which is the absolute value of the difference in angle to port between the two mice, distance b/w mice is the distance between the thoraxes of the mice, velocity (diff) is the absolute value of the difference in velocity between the two mice, and distance to port (diff) which is the absolute value of the difference in distance to reward port between the two mice. **C.** Example frames from each cluster. Colored border matches cluster colors in step 4 depicted in (**A.**). Download Figure 4-2, TIF file.

Next, we asked if social rank differences among competitors influenced behavior, specifically the behavioral states in the unsupervised clusters. Ranks were based on the win/lose outcome of the reward competition, calculated using an Elo score ranking system and simplified into a 1–4 ranking for analysis. During competitions between the most subordinate and dominant mice (rank difference of 3), there were more Cluster 4 behavioral states and fewer Cluster 0 behavioral states (Fig. 4*F*). Lastly, we investigated the strain differences across behavioral states. Cluster 0 was more represented among C57 mice than CD1 mice (*t*_(8)_ = −3.048, adj. *p* = 0.0382; Fig. 4*G*). Interestingly, when the strain was overlaid on the UMAP visualization, there was an area almost exclusively comprised of CD1 data (Fig. 4*H,I*). The area included subsets of Clusters 4, 5, and 7. Further investigation of that area in the plot revealed CD1 mice at the port pressed up against the port wall (Fig. 4*I*), a posture rarely seen among C57 mice. This deeper quantitative analysis using machine learning showed behavioral differences not captured by binary outcomes such as win and loss, possibly uncovering a strain-specific strategy for navigating the reward competition.

### Dominance ranking across assays varies between C57 and CD1 mice

**Figure 5.**
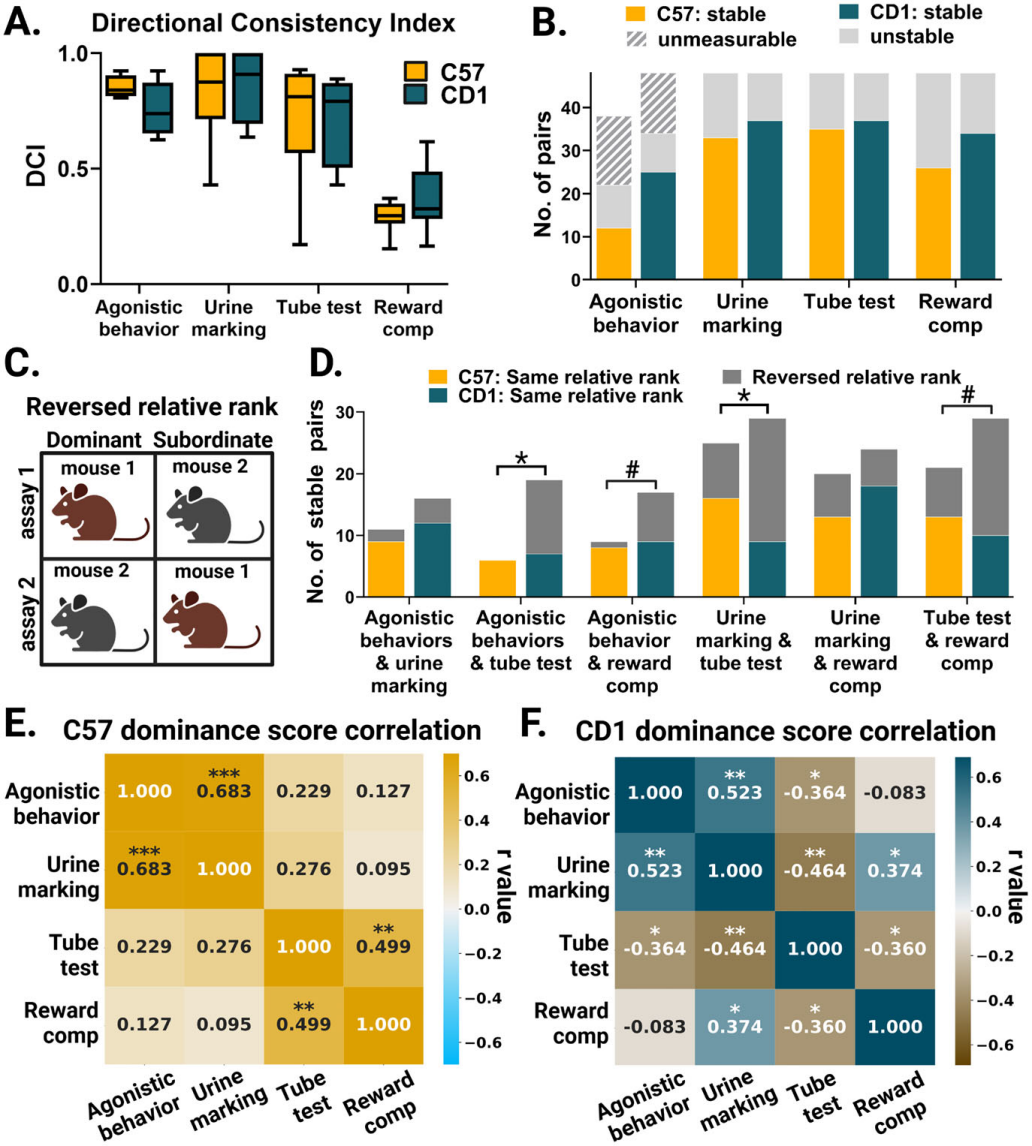
Stability and correlations across assays. ***A***, Directional consistency index means and quartiles plotted for each assay. Unpaired two-tailed *t* tests were applied (agonistic behavior, *n* = 4 cages for C57 and *n* = 7 cages for CD1; all other assays, *n* = 8 cages for both strains). ***B***, Stability of hierarchies across all pairs plotted for each assay. The stability criteria for each assay are described in Materials and Methods. Unmeasurable denotes pairs with no interactions recorded. Agonistic behaviors: *n* = 36 pairs for C57 and 48 pairs for CD1 mice. Urine marking, tube test, and reward competition: *n* = 48 pairs for C57 mice and 48 pairs for CD1 mice. Fisher's exact tests were applied: agonistic behavior, *p* = 0.1614 (excluding unmeasurable pairs); urine marking, *p* = 0.4913; tube test, *p* = 0.8140; reward competition, *p* = 0.1395. ***C***, Schematic of a reversed relative rank pair of mice across Assay 1 and Assay 2, denoted by gray in ***D***. ***D***, Number of pairs that were stable in both assays denoted on the *x*-axis. Yellow denotes stable C57 pairs that maintained relative ranks across both assays, teal denotes stable CD1 pairs that maintained relative ranks, and gray denotes stable pairs that reversed relative ranks. Fisher's exact tests: agonistic behaviors and urine marking, *p* = 0.9999; agonistic behaviors and tube test, *p* = 0.0149; agonistic behaviors and reward competition, *p* = 0.0977; urine marking and tube test, *p* = 0.0278; urine marking and reward competition, *p* = 0.5220; reward competition and tube test, *p* = 0.0848. ***E***, The correlation matrix of Elo scores across assays for C57 mice; *n* = 32 mice for all assays except agonistic behavior which has *n* = 24. Pearson’s correlation *r* value displayed (agonistic behavior vs urine marking, *p* = 0.0002; tube test vs reward competition, *p* = 0.0037). ***F***, Correlation matrix of Elo scores across assays for CD1 mice (*n* = 32). Pearson’s correlation *r* value displayed (Pearson’s correlation *p*-values; agonistic behavior vs urine marking, *p* = 0.0021; agonistic behavior vs tube test, *p* = 0.0403; urine marking vs tube test, *p* = 0.0075; urine marking vs reward competition, *p* = 0.0348; tube test vs reward competition, *p* = 0.0428). *p* < 0.10, *p < 0.05, **p < 0.01, ***p < 0.001, ^#^*p* < 0.1.

Other important considerations for dominance assays are linearity of the hierarchy, stability of ranks, and consistency across dominance metrics. Importantly, we included all mice, regardless of hierarchy stability, and found no correlations between weight and dominance (Extended Data Fig. 5-1). We next measured hierarchy linearity (e.g., if A is dominant over B and B is dominant over C, then A must also be dominant over C). To do this, we used a directional consistency index (DCI) metric that is ideal for small groups (Leiva et al., 2008). There were no strain differences in DCI across all assays (agonistic behavior, *t*_(9)_ = 1.659, *p* = 0.52; urine marking, *t*_(14)_ = 0.3924, *p* = 1.0; tube test, *t*_(14)_ = 0.0054, *p* = 1.00; reward competition, *t*_(14)_ = 1.22, *p* = 0.72). However, there was large variability across assays (Fig. 5*A*), with the reward competition assay showing the lowest DCI levels, suggesting that dominance may not be the only factor influencing behavior during reward competitions. Additionally, we considered potential strain effects on Elo scores. As expected, all assays had significant effects of rank on Elo score. There were no main effects of strain (Extended Data Fig. 5-2*A–D*), but Elo scores for agonistic behaviors had a significant rank and strain interaction (*F*_(3,48)_ = 5.191, *p* = 0.0035; Extended Data Fig. 5-2*A*) where C57 Elo scores showed a more despotic distribution than scores for CD1 mice.

10.1523/ENEURO.0342-24.2024.f5-1 Figure 5-1 **No correlations were found between Elo scores and weight. A-H.** Elo scores plotted against average weight for n = 32 C57 mice for all plots except (**A**) which shows n = 24 C57 mice, and n = 32 CD1 mice for all plots. Linear regression and r value for Pearson correlation are shown in scatterplots. **A.** Pearson correlation for agonistic behavior Elo score and C57 weights: p = 0.2489. **B.** Pearson correlation for agonistic behavior Elo score and weights for CD1: p = 0.6288. **C.** Pearson correlation for urine marking Elo scores and weights for C57: p = 0.5729. **D.** Pearson correlation for urine marking Elo scores and weights for CD1: p = 0.7653. **E.** Pearson correlation for tube test Elo score and weights for C57: p = 0.4537. **F.** Pearson correlation for tube test Elo score and weights for CD1: p = 0.6899. **G.** Pearson correlation for reward competition and weights for C57: p = 0.4122. **H.** Pearson correlation for reward competition and weights for CD1: p = 0.5543. Download Figure 5-1, TIF file.

10.1523/ENEURO.0342-24.2024.f5-2 Figure 5-2 **Elo scores and cross assay correlations. A-D.** Mean Elo score plotted across ranks determined by highest to lowest Elo score. Error bars are standard error of the mean. **A.** Two-way ANOVA; rank: F(3, 48) = 84.06, p < 0.0001; strain: F(1, 48) = 8.108e-11, p = 0.9999; interaction: F(3, 48) = 5.191, p = 0.0035. **B.** Two-way ANOVA; rank: F(3,56) = 83.07, p < 0.0001; strain: F(1,56) = 9.650e-009, p = 0.9999; interaction: F(3,56) = 0.3967, p = 0.7559. **C.** Two-way ANOVA; rank: F(3, 56) = 136.6, p < 0.0001; strain: F(1,56) = 0.006622, p = 0.9354; interaction: F(3,56) = 0.2217, p = 0.8809. **D.** Two-way ANOVA; rank: F(3,56) = 88.49, p < 0.0001; strain: F(1,56) = 1.088e-10, p = 0.9999; interaction: F(3,56) = 1.301, p = 0.2831. **E-N.** Elo scores scatterplots showing individual mice across two assays plotted with a linear regression line and Pearson r value displayed. **E.** Pearson correlation for urine marking and agonistic behaviors Elo scores for C57: p = 0.0002. **F.** Pearson correlation for agonistic behaviors and tube test Elo scores for C57: p = 0.2821. **G.** Pearson correlation for reward competition and tube test Elo scores for C57: p = 0.0037. **H.** Pearson correlation for urine marking and tube test Elo scores for C57: p = 0.1267. **I.** Pearson correlation for reward competition and urine marking Elo scores for C57: p = 0.6056. **J.** Pearson correlation for urine marking and agonistic behaviors Elo scores for CD1: p = 0.0021. **K.** Pearson correlation for agonistic behavior and tube test Elo scores for CD1: p = 0.0403. **L.** Pearson correlation for reward competition and tube test Elo scores for CD1: p = 0.0426. **M.** Pearson correlation for urine marking and tube test Elo scores for CD1: p = 0.0075. **N.** Pearson correlation for reward comp and urine marking Elo scores for CD1: p = 0.0348. For all plots in this figure n = 32 for C57 mice and 32 for CD1 mice except for **A, E & F.** n = 24 C57 mice. **O.** Correlation matrix of David scores across assays for C57 mice; n = 32 mice for all assays except agonistic behavior which has n = 16. Pearson correlation r value displayed (agonistic behavior vs urine marking: p = 0.0220; agonistic behavior vs reward competition: p = 0.0043; tube test vs reward competition: p = 0.0302). **P.** Correlation matrix for David scores across assays for CD1 mice. n = 32 CD1 mice for all assays except agonistic behaviors which had n = 28. Pearson correlation r value displayed (urine marking vs reward competition: 0.0001; tube test vs reward competition: p = 0.0160). **O-P.** David Scores cannot be calculated on cages that did not fight for observation of agonistic behaviors. *p < 0.05, **p < 0.01, ***p < 0.001. Download Figure 5-2, TIF file.

10.1523/ENEURO.0342-24.2024.f5-3 Figure 5-3 **Tube test with reward for CD1 mice. A.** Schematic of a tube test trial in which the winner is rewarded at the end of the tube for pushing the loser mouse out of the tube (rewarded) or not rewarded (non-rewarded). **B-E.** Elo scores across assays. For all plots, n = 16 for rewarded group and n = 17 for non-rewarded group. Linear regressions and Pearson correlations are shown. **B.** Pearson correlation for rewarded tube test and agonistic behavior Elo scores: p = 0.5546. **C.** Pearson correlation for rewarded tube test and urine marking Elo scores: p = 0.1424. **D.** Pearson correlation for non-rewarded tube test and agonistic behavior Elo scores: p = 0.0014. **E.** Pearson correlation for non-rewarded tube test and urine marking Elo scores: p = 0.1160. *p < 0.05, **p < 0.01, ***p < 0.001. Download Figure 5-3, TIF file.

Rank stability was measured and compared across assays, with criteria tailored for each assay (see Materials and Methods). Across assays, there were no strain differences in the number of pairs with stable ranks (Fig. 5*B*). For pairs that had stable social ranks, we quantified the consistency of the relative dominance ranks across assays. Compared to C57 mice, CD1 mice had a greater proportion of pairs that switched relative ranks between the tube test assay and both agonistic behaviors and urine marking assay (Fisher's exact test: agonistic behaviors and tube test, *p* = 0.0149, and urine marking and tube test, *p* = 0.0278). CD1 mice also tended to switch relative ranks between tube test and reward competition but not significantly more than C57 mice (Fisher's exact test, *p* = 0.0848). CD1 mice showed consistency in relative ranks between agonistic behaviors and urine marking as well as urine marking and reward competition. Consistent with previous studies in C57 mice, the relative ranks were consistent for 70% of pairs on average across all assays (Fig. 5*D*). We observed variability across absolute dominant (Rank 1) or absolutely subordinate (Rank 4) mice across assays, which was expected given the reverse ranks seen in CD1 mice in the tube test (Table 8).

**Table 8.** Consistent dominant and subordinate mice per cage across assays

Strain and rank	All assays	Agonistic behaviors and urine marking	Agonistic behavior and tube test	Agonistic behavior and reward competition	Urine marking and tube test	Urine marking and reward competition	Tube test and reward competition
C57 Rank 1	0	3	2	0	2	1	2
C57 Rank 4	2	1	2	3	3	4	4
CD1 Rank 1	0	4	0	2	2	4	1
CD1 Rank 4	0	1	2	1	0	1	0

The values denote the number of mice of that strain and rank (1 being the most dominant and 4 being the most subordinate in a cage) that maintained their rank across any two assays (or all assays) indicated by the column name. For agonistic behavior assay pairs, there were six cages for C57 mice. All other assays had a total of eight cages for CD1 and C57 mice.

Next, we investigated the consistency of dominance rankings across assays for the whole cage. We then quantified the correlation of dominance scores. To measure relative social rank while considering the temporal dynamics of interactions, we used an Elo ranking system to quantify dominance scores for each mouse in each assay (Elo, 1978; Albers and De Vries, 2001). We found strong correlations between dominance scores from agonistic behaviors and urine markings for both strains (C57 *n* = 24, *r* = 0.683, *p* = 0.0002; CD1 *n* = 32, *r* = 0.523, *p* = 0.0021; Fig. 5*E,F*, Extended Data Fig. 5-2*E,J*). However, we found that in CD1 mice, the tube test scores were negatively correlated with dominance scores from all three other assays (tube test vs agonistic behaviors, *r* = −0.364, *p* = 0.0403; tube test vs urine marking, *r* = −0.464, *p* = 0.0075; tube test vs reward competition, *r* = −0.360, *p* = 0.0428; Fig. 5*E,F*, Extended Data Fig. 5-2*K–N*). Importantly, our results were consistent when using a different ranking score, the David score (Gammell et al., 2003; Extended Data Fig. 5-2*O,P*). Our results highlight the variability across dominance assays and across strains and that winning in the tube test for CD1 mice does not correlate with dominance based on territorial marking, agonistic behavior, or competition for rewards.

### Reinforcing the tube testing with rewards does not rescue positive correlations

For CD1 mice, we found a negative correlation across all three assays and the tube test dominance scores. To assess whether this negative correlation was due to a lack of motivation, the tube test was conducted with a sugar pellet reward given to the winner of each trial as originally described in the first tube test publication (Lindzey et al., 1961). Tube test dominance scores were then compared with agonistic behaviors and urine marking dominance scores. The presence of the reward did not result in a positive correlation between the tube test scores and either dominance scores; however, the strong negative correlation seen in the nonrewarded control group was not replicated in the rewarded group (rewarded; tube test vs agonistic behavior, *r* = −0.1597, *p* = 0.5546; tube test vs urine marking, *r* = −0.3837, *p* = 0.1424; nonrewarded; tube test vs agonistic behavior, *r* = −0.7107, *p* = 0.0014; tube test vs urine marking, *r* = −0.3956, *p* = 0.1160; Extended Data Fig. 5-3*B–E*). Our results do not support that reinforcing the tube test with a reward for CD1 mice produces dominance behavior that correlates with other dominance-based assays.

## Discussion

Dominance is a complex behavior that requires many facets of cognition, such as social memory and decision-making (Dwortz et al., 2022). Across species and strains, dominance manifests in distinct ways. Depending on the assay chosen, motor skills, speed, and weight may affect outcomes differently. In this study, we focused on quantifying how dominance behaviors are impacted by strain in male mice. Our results replicate several studies in male C57 mice showing that the tube test and other common dominance assays correlate (Wang et al., 2011; Zhou et al., 2017; Battivelli et al., 2024). Our results demonstrate that outbred CD1 male mice show a larger repertoire of social behaviors with larger differences between winner and loser mice. In addition, we identified that a commonly used dominance assay, the tube test, may not provide consistent results across all mouse strains. Our results highlight that assay choice and design are integral to measuring behavior reliably.

Our study demonstrates CD1 versus C57 strain differences in commonly used dominance assays. Interestingly, we found that in C57 mice, tube test and reward competition dominance scores were strongly correlated. This aligns with our previous results (Padilla-Coreano et al., 2022) showing that reward competition and tube test winning are correlated. However, agonistic behavior dominance scores were not correlated with reward competition dominance scores for C57 or CD1 mice. Comparative analyses of older research show that many species, including mice, do not show high correlations between aggression and access to resources (Uhrich, 1938; Syme, 1974). On the other hand, stable pairwise analysis for C57 mice showed consistency across assays, such that dominant mice in the tube test were also dominant in agonistic behaviors and urine marking, which aligns with other prior published studies (Wang et al., 2011). These results highlight that there may be different facets of dominance that exist orthogonally to each other; within one context, an attribute may elevate an individual's social rank, while in another, it reduces or contributes little to social rank.

Importantly, within one dominance assay, there is a large repertoire of behavioral dynamics. For example, in the tube test trial, a mouse can passively retreat without making any substantial contact with the opponent or the mouse may push and resist the opponent before retreating. In the reward competition, mice may actively gain a dominant position in the reward port by displacing the opponent repeatedly, or a mouse might win the reward while the opponent is disengaged. Using unsupervised, machine learning–based approaches to uncover behavioral states associated with complex behaviors, such as dominance, allows a more nuanced quantification of these behavioral dynamics. Our unsupervised clustering during the reward competition showed behavioral differences across rank and strains not seen through binary win/loss quantification. Moving away from simple, hand-annotated classifications and toward behavioral states that have gradations of competitive behaviors may reveal neural signatures of dominance with greater clarity. Future research studies could employ unsupervised classification of behavioral states to further disentangle neural correlates of rank from competitiveness during dynamic freely moving assays.

Social rank has been shown to affect not only behavior but also neural activity. Manipulation of dopamine neurons in the dorsal raphe nucleus produced greater alterations in social preference in dominant mice compared with intermediate or subordinate mice (Matthews et al., 2016). Social rank also modulates neural activity in response to social odor cues of varying rank and familiarity in mice (W. Lee et al., 2021). Specifically, social competition cues elicited distinct neural responses depending on the relative social rank of the mouse in the same reward competition paradigm used in this study (Padilla-Coreano et al., 2022). Similarly in nonhuman primates, social rank impacts how the amygdala and frontal cortex respond to social cues (Hu et al., 2016; Munuera et al., 2018). In humans, social rank impacts how the prefrontal cortex responds to faces (Ligneul et al., 2017). Taken together, this indicates that rank is a crucial variable that modulates neural computations.

In many dominance studies, social groups that do not form stable hierarchies are removed from further experimental analysis (Varholick et al., 2021). One study using the tube test found stable, linear hierarchies in as little as 19% of groups and found stable despotic hierarchies in 30% of the groups (Varholick et al., 2019). Another study found that roughly 60% of matches produce the same rank from day to day in male C57 mice (Wang et al., 2011). These past studies suggest that mouse hierarchies can range in stability and linearity. Importantly, our study did not have a stability exclusion criterion a priori. Consequently, no assay was the litmus test for stability, which would bias the results toward subjects who behave in a certain way in that assay. The inclusion of all subjects for cross-assay correlations implies that any correlations we found are maintained even with less stable hierarchies. Nonetheless, a caveat of our findings is that it may be limited to male mice. Although some research has shown that females can perform the tube test (Garner et al., 2004; Garfield et al., 2011; van den Berg et al., 2015) and display agonistic behaviors, social hierarchy literature is still biased toward males (Fulenwider et al., 2021). Some complications with measuring social rank in females include that the most observable agonistic behavior in C57 female mice is barbering (Garner et al., 2004; Bechard et al., 2011) and that female CD1 mice have lower levels of aggression as compared with males (Bartolomucci et al., 2004). Territorial urine marking has only been reported in breeding females in one study (Hurst, 1990b), and stable social ranks have been noted in a large, naturalistic vivarium (Hurst, 1987), both of which limit how social rank can be measured in female mice.

Dominance metrics can be divided into two main types: resource competition and agonistic behaviors (Fulenwider et al., 2021). Tube test, urine marking, and ultrasonic vocalizations can be described as competition for territory and mates and fall under resource competition. Most notably, we demonstrated that in CD1 mice, tube test dominance had a negative correlation with all three other dominance scores. This negative correlation was not rescued by reinforcing the tube using a reward, suggesting that the original CD1 tube results are not due to lack of motivation within the tube test paradigm. Another study showed positive correlations between tube test results and food access in CD1 mice, suggesting that the exact food resource competition assay may impact results significantly (Y-A. Lee and Goto, 2018). Additionally, the first tube test paradigm was done in food-restricted mice using a reward (Lindzey et al., 1961, 1966). Our results in CD1 mice align with the findings of Lindzey et al. (1966) who reported that when two inbred mouse lines compete, there are correlations between aggression and food competition dominance but negative correlations between winning in the tube test and other dominance assays. However, agonistic behaviors have been shown to correlate with tube test winning in C57 male mice, which is consistent with our pair rank data and our rank correlations (Wang et al., 2011; Larrieu et al., 2017). However, not all inbred strains exhibit this correlation between agonistic behaviors and winning in the tube test (Lindzey et al., 1966; Barabas et al., 2021). Even in C57 male mice, there are studies showing opposing relationships between barbering behavior and winning in the tube test (Garner et al., 2004; Wang et al., 2011). Thus, our findings and the previous literature illustrate that resource competition and agonistic behaviors may have different biological mechanisms impacted by the genetics of different mouse strains.

Our results support that dominance behaviors are impacted by genetics. Given our findings, investigators who measure dominance in laboratory mice should pay close attention to potential strain effects. While the tube test may be an optimal assay for male C57 mice, the tube test may not reliably measure dominance hierarchy in CD1 males.

## Synthesis

Reviewing Editor: Sam Golden, The University of Washington Decisions are customarily a result of the Reviewing Editor and the peer reviewers coming together and discussing their recommendations until a consensus is reached. When revisions are invited, a fact-based synthesis statement explaining their decision and outlining what is needed to prepare a revision will be listed below. The following reviewer(s) agreed to reveal their identity: Brian Trainor.

Dear Dr. Padilla-Coreano,

Your manuscript has been reviewed by two experts in the field. Both reviewers commend the manuscript for its well-designed study and clear writing, highlighting its potential contribution to the field of behavioral neuroscience. The reviewers and myself agree that the manuscript is appropriate for publication following minor revision and re-review. Below you will find my synopsis of the key reviewer comments, along with the full reviewer comments. Please address each reviewer comment point by point. I look forward to handling the resubmission.

Reviewer 1 appreciates the use of SLEAP to analyze dominance behaviors in mice, and the inclusion of relevant literature. However, concerns are raised about the variability in observation times and its impact on scoring aggressive behaviors. They suggest further clarification on the SLEAP analysis and recommend addressing specific issues such as the age of the mice during testing and potential differences in food restriction between dominant and subordinate animals. Reviewer 2 echoes the positive feedback on the study's design and writing but offers specific recommendations for the antagonistic behavior analysis. They suggest either shortening the observation period to the first 15 minutes or analyzing behavior across smaller time bins to better understand the relationship between attack patterns and dominance hierarchy. Additionally, they request clarification on whether the mice in the antagonistic interaction tests were cage mates or novel animals, and they ask for more details on the hyperparameters used in the UMAP analysis.

Overall, the manuscript was positively received by the reviewers and the requested revisions will improve the final manuscript.

Reviewer 1:

In this manuscript the authors perform a number of behavioral assays to assess dominance status in two commonly used strains of mice. The authors emphasize the importance of considering multiple factors when testing for dominance behavior in commonly studied mouse strains. These factors include genetic background, use of appropriate behavioral assays, and proper scoring of dominant behaviors. The authors also use a supervised machine learning tool called "SLEAP" to analyze video data and make predictions about specific aggressive behaviors in both CD1 and C57 mice. The paper is well written and included both modern and older literature relevant to the studies described. Overall, with relatively minor revisions this manuscript should make a nice contribution to eNeuro.

Major comments

There was a significant amount of variation in observation time between cages/conditions. Having a window of analysis from "at least 15 minutes and up to 45 minutes in a session," for example, is widely variable and could impact the way aggressive behaviors were scored. The authors should address how this was controlled for in the methods.

The introduction emphasizes the potential of SLEAP as a way to categorize and predict behavior. However, the discussion does not make it obvious how these analyses led to novel insights. Most of the data addressed in the discussion address hand scored analyses. It would be great if the authors could explain specific insights that were gained from the SLEAP analysis.

Can the authors please explain how clusters 4, 5 and 7 are "almost exclusively comprised of CD1 data" yet figure 4G shows that for 5 and 7 a similar % frames per recording are displayed for the two strains. Cluster 4 also has a fair number of frames listed for C57.

Food restriction: is it possible that dominant animals were less restricted than subordinates? Maybe the dominants are more competitive for food in the home cages?

Minor comments

The authors could connect the examples of dominance behaviors in birds and primates a bit more to the mouse studies.

Although it is noted that all mice were "8 weeks old" upon arrival at the lab, there is no information on the age at which tests were actually conducted. Age information is important and relevant, especially when testing aggression in mice species.

Typo: "Mice we observed in a dimly lit and quiet room for at least 15 minutes and up to 45 minutes in a session." (p. 3)

Varholick et al, 2021 is missing citation information.

Did the latency to traverse the tube in rewarded tests decrease during training?

It would be useful to include trial duration means/std errors in a table for investigators who may try to replicate these studies.

Reviewer 2:

This is a well written paper and a well designed study.

With regard to the antagonistic behavior analysis, I have found in my own work that animals tend to cluster into early or late attackers, and have often wondered about the significance of this. I would suggest a) either shortening all of your trials to the first 15 minutes of observation and using only these data, and/or b) evaluating antagonistic behavior across smaller time bins in your assays and looking at relationships between attack distribution and hierarchy position. It seems likely that more dominant animals may have different attack patterns than less dominant animals. Also please clarify whether the mice in the antagonistic interaction tests were cage mates or novel animals.

For your UMAP analysis, please add details on your hyperparameters for the models and how you chose them.

## Author Response

Thank you to the reviewers for acknowledging our rigorous experimental design. We appreciate the feedback and call for clarity on certain sections. We have revised the data included in our agonistic behavior observations for both Figure 1 and Figure 1-1. For these figures, all data points now come from sessions of equal length. As per the reviewer's comment, we believe this addresses potential differences in fight distributions within a session. We have also added more details regarding the methodology of the tube test and the rewarded tube test as well as an added panel in Figure 3 regarding tube test trial lengths. Lastly, we have clarified the hyperparameters used during the UMAP analysis and the significance of our behavioral differences found from the unsupervised clustering techniques. We feel these changes have strengthened our paper and hope that comments and concerns have been adequately addressed.

Reviewer 1:

In this manuscript the authors perform a number of behavioral assays to assess dominance status in two commonly used strains of mice. The authors emphasize the importance of considering multiple factors when testing for dominance behavior in commonly studied mouse strains. These factors include genetic background, use of appropriate behavioral assays, and proper scoring of dominant behaviors. The authors also use a supervised machine learning tool called "SLEAP" to analyze video data and make predictions about specific aggressive behaviors in both CD1 and C57 mice. The paper is well written and included both modern and older literature relevant to the studies described. Overall, with relatively minor revisions this manuscript should make a nice contribution to eNeuro.

Major comments There was a significant amount of variation in observation time between cages/conditions. Having a window of analysis from "at least 15 minutes and up to 45 minutes in a session," for example, is widely variable and could impact the way aggressive behaviors were scored. The authors should address how this was controlled for in the methods.

We thank the reviewer for bringing up this important point. Our initial goal was to use as much data as possible, however, this made the interpretation of our original frequency metric difficult. We have excluded the handful of observations that only were done for 15 minutes, and now we uniformly report the number of agonistic behaviors in 30 minutes. We believe that this makes interpretation of the results and replicating the experiments easier. Similarly, we have only included social opportunity manipulations that were 30 minutes of observations without the dominant mouse and 15 minutes of observation after its return. Importantly in our original submission we showed the average over multiple sessions of various lengths for data in Fig 1-1, but to increase rigor we are showing the first social opportunity manipulation per mouse. We believe this will make it easier for future studies to replicate. Importantly, this standardization affected a small portion of our data and did not change our conclusions that CD1 mice show more agonistic behaviors. We updated figures, legends, methods and result sections to reflect the new analyses we have done.

New methods descriptions: "Mice were observed in a dimly lit and quiet room for 30 minutes. Interactions between cagemates were observed by trained observers. Fights and chases along with initiator and receiver were recorded. All cages included in Figure 1 were observed for at least 6 sessions and the mean fights and chases plots represent the average per mouse across all sessions. A subset of cages was subject to social opportunity manipulations. For the social opportunity condition, the mouse who had initiated the most fights prior to the vacuum session was removed into a separate clean cage. If no fights had ever been initiated in a given cage, a mouse was chosen at random for removal. After 30 minutes of observation without the removed mouse, the mouse was returned to its homecage, and observations were recorded for an additional 15 minutes." (last paragraph on page 2) "A two-tailed Fisher's exact test was applied to all initiated/received fights and chases proportions. Average fights and average chases per 30 minute session were calculated per mouse (total fights OR chases/number of sessions) and then a two-tailed Mann-Whitney U test was applied to the averages. To facilitate interpretation of data shown in Fig. 1, these calculations did not include any data from social opportunity manipulations and included only sessions of 30 minutes of observation. For Fig. 1-1, data calculations were done on data from a single social opportunity manipulation which included 30 minutes of observation time without the separated mouse and 15 minutes of observations immediately after the separated mouse was returned to the homecage." (First paragraph on page 6) New language in results section "We first quantified dominance metrics based on agonistic behavioral observations from cages of four mice. For both strains, we observed interactions in the homecage for at least 5 sessions ranging from 15-30 minutes, noting who initiated and who received fights and chases (Fig. 1A). Although more CD1 mice initiated and received fights, no significant strain differences were observed (Fig. 1B, C). Consistent with previous reports (Hsieh et al., 2018), CD1 mice, on average, fought more per observation session than C57 mice (U = 175, p = 0.0137, Fig. 1D). No significant differences were observed in chasing behavior (Fig. 1E-G.) Previous research shows that across species (Maruska et al., 2013; Williamson et al., 2017), individuals quickly increase agonistic behavior during a social opportunity, i.e., when the top-ranking individual is not present. To quantify sensitivity to social opportunity in laboratory standard cages in C57 and CD1 mice, we removed the mouse that initiated the most fights from each cage and observed conspecific behavior both in their absence and after returning them to the cage (Fig. 1-1A). During social opportunity manipulations, CD1 mice exhibited more fighting behavior per session as well as having a greater proportion of mice initiating fights compared to C57 mice. However, these differences were not significant, suggesting larger sample sizes are needed to observe these social opportunity differences in the lab setting (p=0.052 and p=0.067; Fig. 1-1B,D). These data show that CD1 mice exhibit more agonistic behavior than C57 mice and suggests that C57 mice may be less responsive to social opportunities than CD1 mice." (2nd and 3rd paragraphs, Page 7) The updated figures 1 and 1-1 are below:

Figure 1. CD1 mice showed more agonistic behaviors in their homecage. A. Schematic of agonistic behavioral events recorded: fighting (left) and chasing (right). B. Percent of mice that initiated at least one fight during any observation (Fisher's exact test, p = 0.1468). C. Percent of mice that received at least one fight during any observation (Fisher's exact test, p = 0.2124). D. Average number of fights in a 30 minute observation session per mouse (Mann-Whitney U = 175, p = 0.0137) E. Percent of mice that initiated at least one chase across all observations (Fisher's exact test, p =0.5612). F. Percent of mice that received at least one chase across all observations (Fisher's exact test, p = 0.1930). G. Average number of chases in a 30 minute observation session (Mann-Whitney U = 226.5, p = 0.1885). For all plots, n=24 C57 and n=24 CD1. Plots show mean and error bars show standard error of the mean. *p<0.05, **p<0.01, ***p<0.001.

Figure 1-1. Social opportunity's effect on agonistic behaviors differs across strains. A. Schematic of behavioral paradigm: 1) mouse who initiated the most fights was identified and placed into a separate cage. 2) observation of agonistic behaviors was recorded without the dominant mouse for 30 minutes. 3) The separated mouse was returned to the homecage, and agonistic behaviors were recorded for an additional 15 minutes. Data from observations during 2) removal of dominant and 3) return of the dominant were combined for 45 minutes of analyzed recordings. B. Percent of mice that initiated at least one fight during the observations (Fisher's exact test, p = 0.0674). C. Percent of mice that received at least one fight during the observation (Fisher's exact test, p = 0.1523). D. Number of fights in the 45-minute sessions per mouse (Mann-Whitney U = 114, p = 0.0521). E. Percent of mice that initiated at least one chase during the observations (Fisher's exact; p = 0.2714). F. Percent of mice that received at least one chase during the observation (Fisher's exact; p = 0.4774). G. Number of chases during the 45-minute session (Mann-Whitney U = 128.5, p = 0.1795). Plots show mean and error bars show standard error of the mean. For all plots, n=12 C57 and n=28 CD1. *p<0.05, **p<0.01, ***p<0.001, # p<0.1.

The introduction emphasizes the potential of SLEAP as a way to categorize and predict behavior. However, the discussion does not make it obvious how these analyses led to novel insights. Most of the data addressed in the discussion address hand scored analyses. It would be great if the authors could explain specific insights that were gained from the SLEAP analysis.

We thank the reviewer for this insightful comment. We agree that further discussion is warranted. The differences in behavior found using SLEAP provide proof of concept, that unsupervised behavior analysis can uncover differences that binary labels of wins and losses could not. The scope of our paper does not attempt to make any claims about the significance of this CD1-specific posture beyond that it is a strategy that is not seen in C57 mice. The introduction, results and discussion have been edited to hopefully make clear that it is the potential usefulness of these methodologies to identify behavioral differences that are missed by traditional approaches that is significant.

Introduction page 2, 2nd paragraph reads: "Unsupervised clustering on the competition data revealed differences across strains and rank that were not detectable with traditional behavioral analysis, potentially highlighting a CD1-specific strategy for the reward competition task." The results section, page 8, 2nd paragraph now reads: ""First, to track behavior with more granularity than human annotation, we used SLEAP (Pereira et al., 2022), a deep learning tool for pose estimation, to track the positions of both mice during the reward competition. This deeper quantitative analysis using machine learning showed behavioral differences not captured by binary outcomes such as win and loss, possibly uncovering a strain-specific strategy for navigating the reward competition." And lastly the discussion on page 10, 2nd paragraph reads: "Future research studies could employ unsupervised classification of behavioral states could be used to further disentangle neural correlates of rank from competitiveness during dynamic freely moving assays." Can the authors please explain how clusters 4, 5 and 7 are "almost exclusively comprised of CD1 data" yet figure 4G shows that for 5 and 7 a similar % frames per recording are displayed for the two strains. Cluster 4 also has a fair number of frames listed for C57.

We apologize for the lack of clarity. We have revised the descriptions of Figure 4H and I to address the reviewer's suggestion.

The results section on page 8, 1st paragraph now reads: "Interestingly, when strain was overlaid on the UMAP visualization, there was an area almost exclusively comprised of CD1 data (Fig. 4H, I). The area included subsets of clusters 4, 5 and 7. Further investigation of that area in the plot revealed CD1 mice at the port pressed up against the port wall (Fig. 4I), a posture rarely seen among C57 mice. This deeper quantitative analysis using machine learning showed behavioral differences not captured by binary outcomes such as win and loss, possibly uncovering a strain-specific strategy for navigating the reward competition." Food restriction: is it possible that dominant animals were less restricted than subordinates? Maybe the dominants are more competitive for food in the home cages? We thank the reviewer for bringing up this possibility. We reviewed our data considering weight changes. These new analyses are now shown in Figure 4-1. The average weight lost due to food restriction and competition dominance score does not correlate. This may be because total weight doesn't consider body composition (fat vs muscle) and there may be dominant mice who are leaner than subordinates. These results are discussed in results in the 1st paragraph on page 8. The new figure is included below:

Figure 4-1. Weight loss is not correlated with reward competition dominance. A. Average weight loss in grams across reward competition days for C57 mice (n=32) plotted against their final Elo score for reward competition (Pearson correlation r=0.015, p = 0.9145). B. Average weight loss in grams across both competition days for CD1 mice (n=32) plotted against their final Elo score for reward competition (Pearson correlation r=-0.114, p = 0.5338).

Minor comments The authors could connect the examples of dominance behaviors in birds and primates a bit more to the mouse studies.

Thank you for this suggestion. Although we commented on different displays of dominance in the introduction it is equally important that dominance displays are conserved across species as well. The following sentence and references have been added into the first paragraph of the introduction on page 1: "Some displays of dominance are conserved across species, such as linear hierarchies in chickens and mice (Rushen, 1982; Wang et al., 2011) or primary access to limited resources for dominant macaques and rats (Boccia et al., 1988; Costa et al., 2021)." Although it is noted that all mice were "8 weeks old" upon arrival at the lab, there is no information on the age at which tests were actually conducted. Age information is important and relevant, especially when testing aggression in mice species.

This is a really important point for rigor and future reproducibility. To address this concern, we now include table 2, which is cited in the Animals section under Methods. This table includes the ages of the mice during each assay.

Cohort Agonistic Behaviors Urine Marking Tube Test Reward Competition Cohort 1 12-15 weeks 14-16 weeks 9-12 weeks 12-14 weeks Cohort 2 10-11 weeks 12 weeks 11-14 weeks 13-14 weeks Cohort 3 11-14 weeks 12 weeks 10-14 weeks 13-15 weeks Table 2. Cohort Ages. Ages of mice during each behavioral assay across all 3 cohorts.

Typo: "Mice we observed in a dimly lit and quiet room for at least 15 minutes and up to 45 minutes in a session." (p. 3) We have corrected this typo.

Varholick et al, 2021 is missing citation information.

We have fixed this missing citation.

Did the latency to traverse the tube in rewarded tests decrease during training? Yes, latency to traverse the tube decreases across training days. Additional methodological details have been added to the reward tube test section in the methods. Unfortunately, we did not record videos during tube training, however historical observations in the lab, and statements in the protocol paper from Dr. Hailan Hu's lab, indicates that across days of tube training the latency to transverse the tube does decrease: "It becomes quicker and easier for the mice to go through the tube. ...Steps 2-11, training mice to go through the tube: 2 d, ~15 min per mouse on day 1 and ~10 min per mouse on day 2" (Fan et al. Using the tube test to measure social hierarchy in mice, Nature Protocols, 2019).

We have added the following details in the tube test section of the methods to aid in reproducibility:

The first paragraph on page 3 now has the sentence: "A plastic cylinder, slightly smaller than the tube, was used to stop mice from retreating or to encourage them to move forward by gently contacting the back of the mouse." The 2nd paragraph on page 3 of the methods now reads: "In the rewarded tube test, 31 male CD1 mice, aged 8 weeks upon arrival, were used. All mice were food-restricted to 85% of their body weight, with 16 mice for the experimental (rewarded) group and 17 for the control (non-rewarded) group ran in two cohorts. Mice in the rewarded group were introduced to sugar pellets in their home cages before training to familiarize them with the new food. Individual training lasted 4-5 days with the sugar pellet or no pellet for controls was performed, consistent within a cohort. During training trials, a sugar pellet was placed on the table at the exit of the tube so that the subject could retrieve it upon exiting the tube. Training continued until each mouse ate 8 pellets to ensure they associated reaching the end of the tube with receiving the pellet. The pellet's distance was adjusted based on each mouse's speed and motivation. For the trial during the first day of training, the sugar pellet was placed right at the exit of the tube. For the following days of training the position of the pellet was adjusted based on the individual mouse performance. For mice with longer latencies to traverse the tube, the pellet was placed at the tube's exit, while for mice that were crossing the tube quicker the pellet was progressively farther away every trial, up to a maximum of 4 centimeters to reflect tube testing conditions. As performance improved, the distance was increased by 1-centimeter increments, with the distance adjusted either closer or farther for each of the 8 trials depending on the mouse's success in retrieving the previous pellet. For the control group, training followed the same procedure without providing the reward, and mice were paired for matches against their cagemates. Each mouse within a pair underwent identical tube training. Each cage was assigned either experimental or control and matches were only against cagemates. Therefore, each mouse within a match was subjected to the same method of tube training. In the rewarded tube test, once a mouse was pushed out of the tube, the loser was removed while simultaneously placing a sugar pellet 4 centimeters from the exit for the winner mouse." It would be useful to include trial duration means/std errors in a table for investigators who may try to replicate these studies.

To facilitate replications, we have added figure 3B showing trial duration for tube test matches in C57 vs CD1 mice. Unfortunately, we did not record video during the rewarded tube test sessions, so we are unable to report the trial latencies for the rewarded experiments. The new updated figure is here:

Figure 3. Tube test behaviors show minor strain differences. A. Schematic of a tube test trial in which the loser is pushed out of the tube by the winner. B. Mean trial duration for match length for C57 mice (mean = 15.46 sec, SEM = 1.415 sec) and CD1 mice (mean = 18.39 sec, SEM = 2.033). C-E. Average percentage of a trial spent in the given behavior is shown by trial outcome and by strain. Winner (black) and loser (grey) averages were calculated from all data, while C57 (yellow) and CD1 (teal) averages were calculated combining winners and losers. C. Percent tube trial time spent pushing. Winner vs loser: t(266) = -3.508, p = 0.0005. C57 vs CD1: t(45) = -2.600, p = 0.0126. D. Percent tube trial time spent resisting. Winner vs loser: t(264) = 0.814, p = 0.463. C57 vs CD1: t(44.9) =-2.662, p = 0.0107. E. Percent tube trial time spent retreating in contact. Winners vs losers: t(263) = 7.695, p < 0.0001. C57 vs CD1: t(45.5) = -0.579, p = 0.5656. F. Percent tube trial time spent passively retreating (without contact from competitor). Winners vs losers: t(263) = 1.456, p < 0.0001. C57 vs CD1: t(44.8) = 1.456, p = 0.1524. Plots include data for n=24 C57 mice and n=24 CD1 mice across 144 trials; n=72 C57 trials, and n=72 CD1 trials. Plots show mean and error bars show standard error of the mean. All t-tests were calculated using estimated marginal means of a mixed effects linear model, reported p values were adjusted for multiple comparisons using the Holm-Bonferroni method. *p<0.05, **p<0.01, ***p<0.001.

Reviewer 2:

This is a well written paper and a well designed study.

With regard to the antagonistic behavior analysis, I have found in my own work that animals tend to cluster into early or late attackers, and have often wondered about the significance of this. I would suggest a) either shortening all of your trials to the first 15 minutes of observation and using only these data, and/or b) evaluating antagonistic behavior across smaller time bins in your assays and looking at relationships between attack distribution and hierarchy position. It seems likely that more dominant animals may have different attack patterns than less dominant animals. Also please clarify whether the mice in the antagonistic interaction tests were cage mates or novel animals.

Unfortunately, we did the observations by hand, as a video camera could not capture all the mice without occlusion since the cage top was on the cage. Humans were more capable of dynamically moving to see all mice. As we explained in reviewer 1 response, our initial goal was to use as much data as possible, however we recognize this made the interpretation of our metric more difficult. We have excluded the handful of observations that only were done for 15 minutes, and now we uniformly report the number of agonistic behaviors in 30 minutes. We believe that this makes interpretation of the results and replicating the experiments easier. The figure, legend, methods and result sections have been updated to reflect the analyses we have done.

The last paragraph on page 2 in the methods section now reads: "Interactions between cagemates were observed by trained observers" and the second paragraph of the results on page 7 now says, "For both strains, we observed interactions amongst cagemates in the homecage for at least 6 sessions of 30 minutes, noting who initiated and who received fights and chases." For your UMAP analysis, please add details on your hyperparameters for the models and how you chose them.

We thank the reviewer for bringing this point up. We have added additional details on how we chose hyperparameters for the UMAP analysis. Importantly, we only use UMAP for visualization of the data, not for clustering. Our methods section now reads as follows in page 4, second paragraph: "Trained observers watched videos constructed of 600 randomly selected frames from each cluster (Supp. Videos 1-8). The number of clusters was decided based on the interpretability of the resulting videos, using the maximum number of clusters that resulted in videos that could be uniquely described by the trained observers. UMAP dimensionality reduction was applied for visualization after clustering. UMAP.fit_transfom() from the python package umap-learn, version 0.5.5, was used. Default parameters were used except for random_state, which was defined as 42 to avoid stochasticity during plotting. Importantly, the dimensionality reduction was just for visualization and the clustering was done using all 7 features, therefore the dimensionality reduction has no effects on cluster assignments."
