# Supplementary figures and images for: A Multiparadigm Approach to Characterize Dominance Behaviors in CD1 and C57BL6 Male Mice
Source: eNeuro. 2024 Nov 19;11(11):ENEURO.0342-24.2024. doi: 10.1523/ENEURO.0342-24.2024 (PMC11599796; doi:10.1523/ENEURO.0342-24.2024)

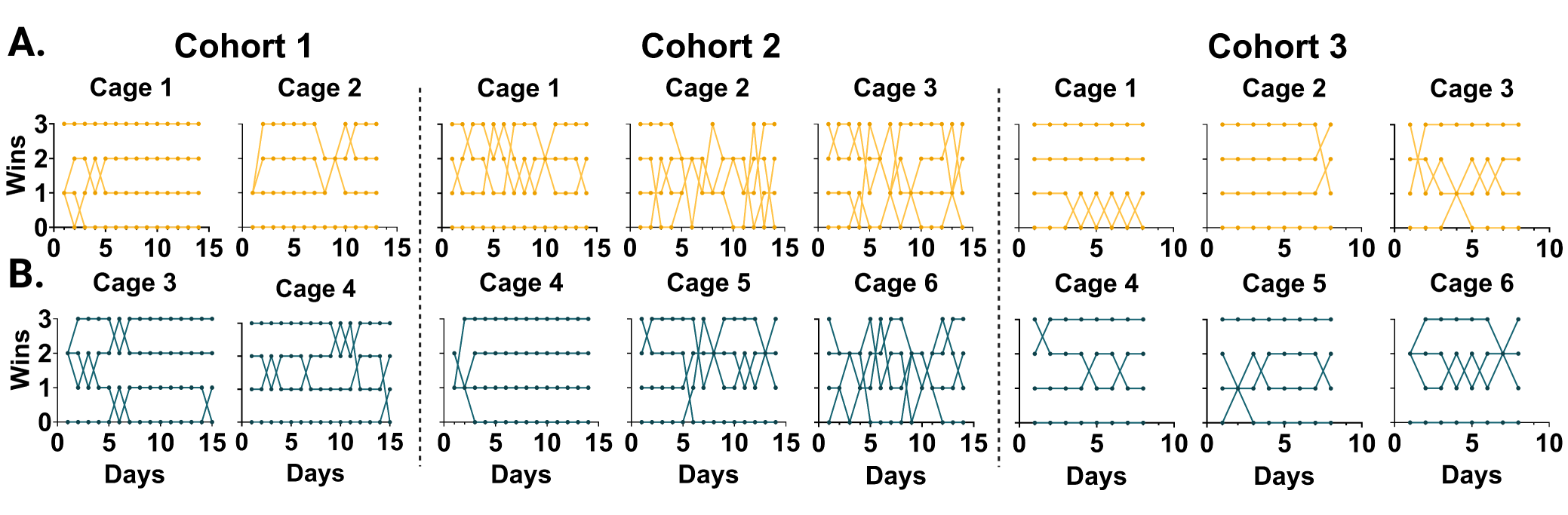

Supplement: Figure 3-1 — Tube test stability across cohorts. A. C57BL6 (yellow) cages across the three cohorts. Each colored line represents a mouse, and each point represents the total number of wins per day. B. CD1 (teal) cages across the three cohorts. Each colored line represents a mouse, and each point represents the total number of wins for that day. For all plots, there were four mice per cage and each mouse was subjected to three matches per day. Thin dashed lines separate the cohorts. Download Figure 3-1, TIF file. [file eneuro-11-ENEURO.0342-24.2024-s002.tif]

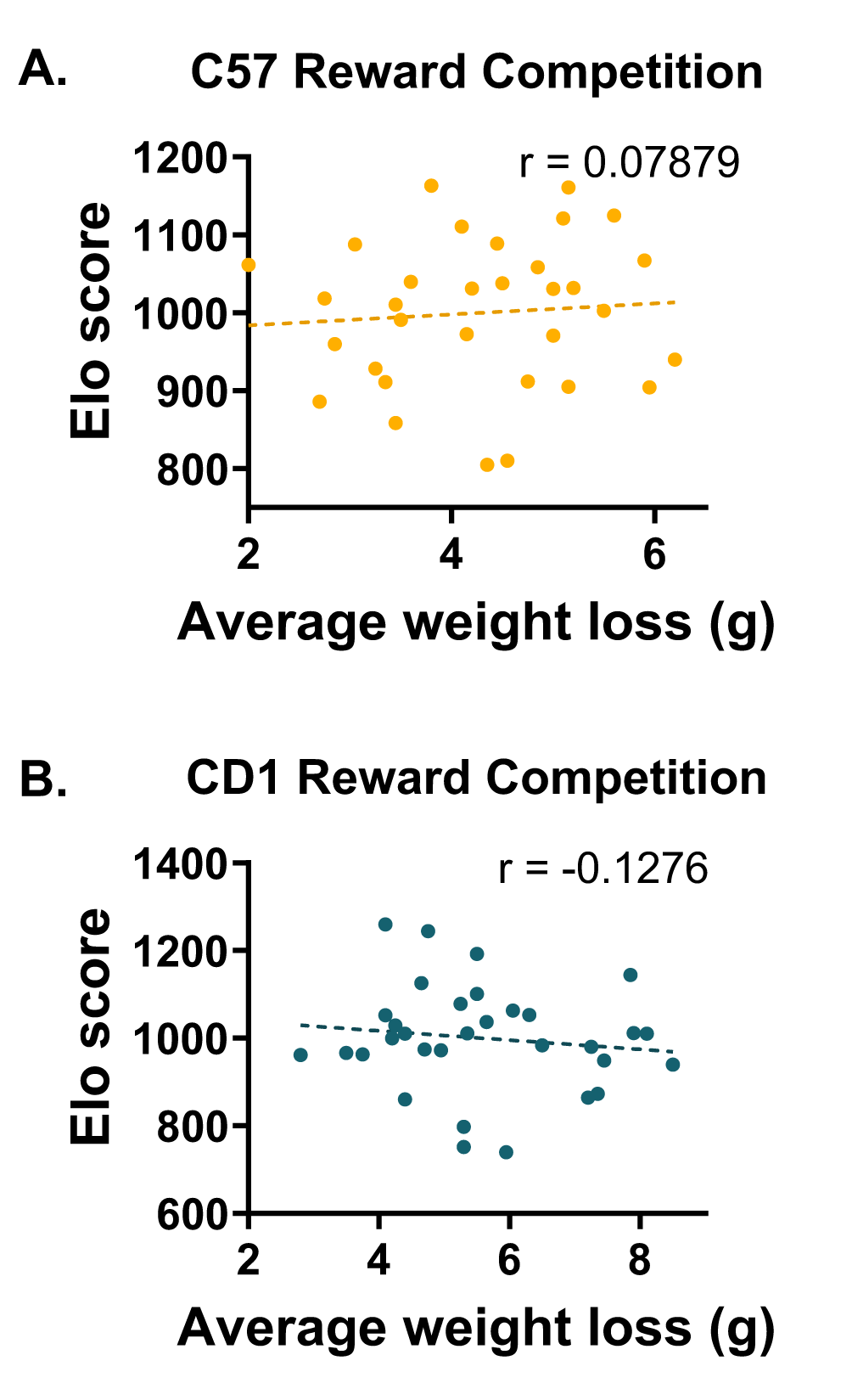

Supplement: Figure 4-1 — Weight loss is not correlated with reward competition dominance. A. Average weight loss in grams across reward competition days for C57 mice (n = 32) plotted against their final Elo score for reward competition (Pearson correlation r = 0.015, p = 0.9145). B. Average weight loss in grams across both competition days for CD1 mice (n = 32) plotted against their final Elo score for reward competition (Pearson correlation r = -0.114, p = 0.5338). Download Figure 4-1, TIF file. [file eneuro-11-ENEURO.0342-24.2024-s003.tif]

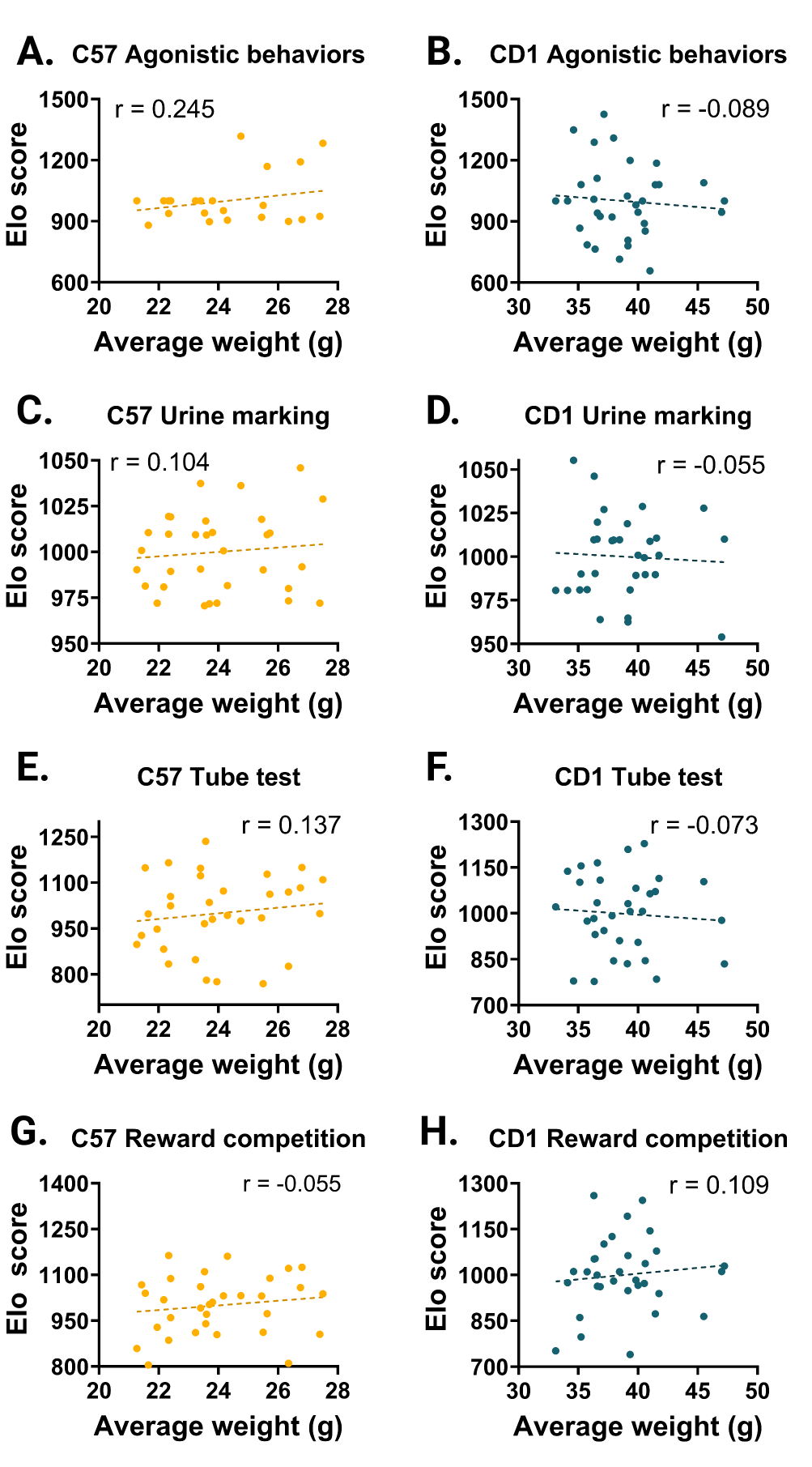

Supplement: Figure 5-1 — No correlations were found between Elo scores and weight. A-H. Elo scores plotted against average weight for n = 32 C57 mice for all plots except (A) which shows n = 24 C57 mice, and n = 32 CD1 mice for all plots. Linear regression and r value for Pearson correlation are shown in scatterplots. A. Pearson correlation for agonistic behavior Elo score and C57 weights: p = 0.2489. B. Pearson correlation for agonistic behavior Elo score and weights for CD1: p = 0.6288. C. Pearson correlation for urine marking Elo scores and weights for C57: p = 0.5729. D. Pearson correlation for urine marking Elo scores and weights for CD1: p = 0.7653. E. Pearson correlation for tube test Elo score and weights for C57: p = 0.4537. F. Pearson correlation for tube test Elo score and weights for CD1: p = 0.6899. G. Pearson correlation for reward competition and weights for C57: p = 0.4122. H. Pearson correlation for reward competition and weights for CD1: p = 0.5543. Download Figure 5-1, TIF file. [file eneuro-11-ENEURO.0342-24.2024-s005.tif]

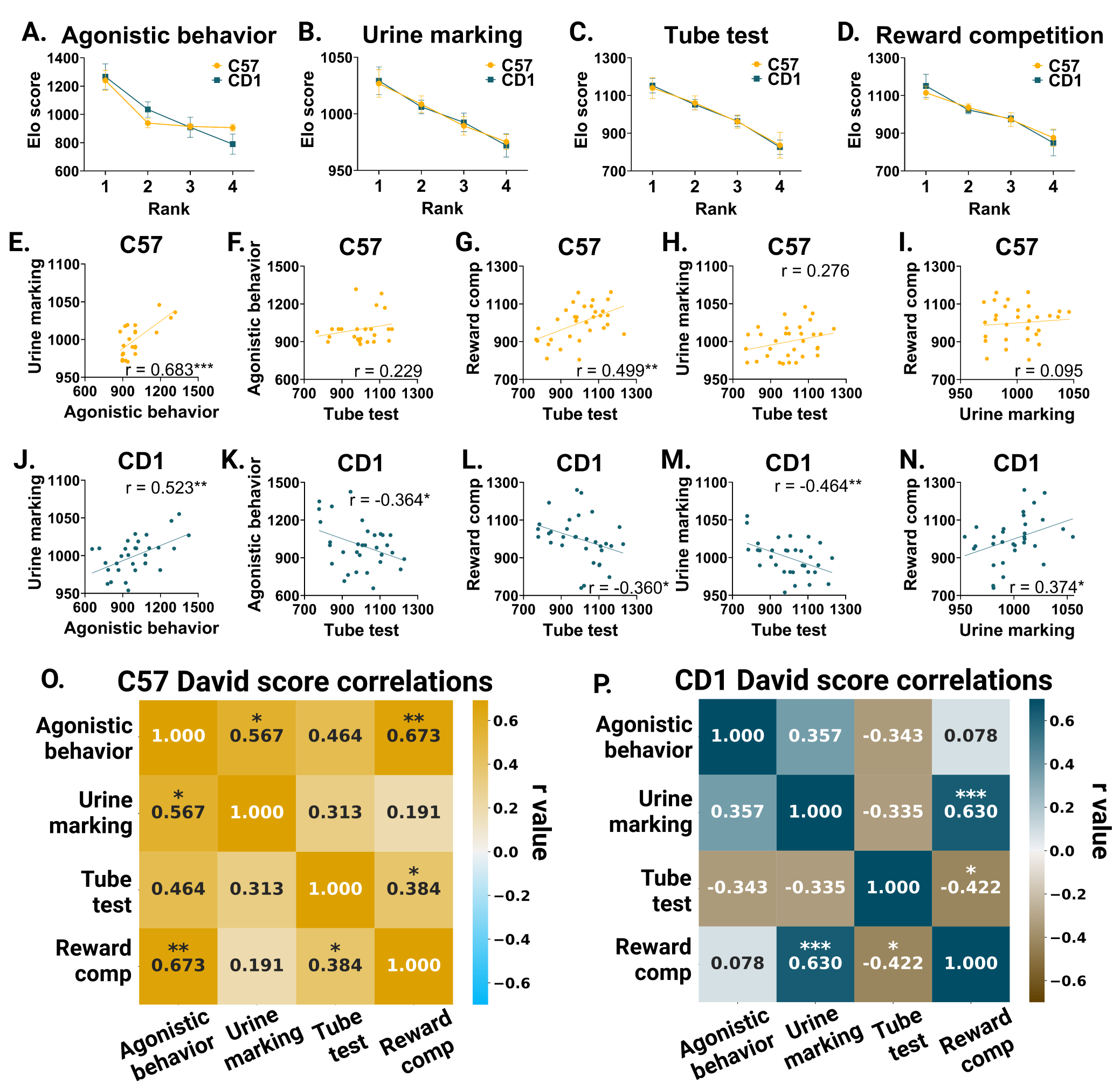

Supplement: Figure 5-2 — Elo scores and cross assay correlations. A-D. Mean Elo score plotted across ranks determined by highest to lowest Elo score. Error bars are standard error of the mean. A. Two-way ANOVA; rank: F(3, 48) = 84.06, p < 0.0001; strain: F(1, 48) = 8.108e-11, p = 0.9999; interaction: F(3, 48) = 5.191, p = 0.0035. B. Two-way ANOVA; rank: F(3,56) = 83.07, p < 0.0001; strain: F(1,56) = 9.650e-009, p = 0.9999; interaction: F(3,56) = 0.3967, p = 0.7559. C. Two-way ANOVA; rank: F(3, 56) = 136.6, p < 0.0001; strain: F(1,56) = 0.006622, p = 0.9354; interaction: F(3,56) = 0.2217, p = 0.8809. D. Two-way ANOVA; rank: F(3,56) = 88.49, p < 0.0001; strain: F(1,56) = 1.088e-10, p = 0.9999; interaction: F(3,56) = 1.301, p = 0.2831. E-N. Elo scores scatterplots showing individual mice across two assays plotted with a linear regression line and Pearson r value displayed. E. Pearson correlation for urine marking and agonistic behaviors Elo scores for C57: p = 0.0002. F. Pearson correlation for agonistic behaviors and tube test Elo scores for C57: p = 0.2821. G. Pearson correlation for reward competition and tube test Elo scores for C57: p = 0.0037. H. Pearson correlation for urine marking and tube test Elo scores for C57: p = 0.1267. I. Pearson correlation for reward competition and urine marking Elo scores for C57: p = 0.6056. J. Pearson correlation for urine marking and agonistic behaviors Elo scores for CD1: p = 0.0021. K. Pearson correlation for agonistic behavior and tube test Elo scores for CD1: p = 0.0403. L. Pearson correlation for reward competition and tube test Elo scores for CD1: p = 0.0426. M. Pearson correlation for urine marking and tube test Elo scores for CD1: p = 0.0075. N. Pearson correlation for reward comp and urine marking Elo scores for CD1: p = 0.0348. For all plots in this figure n = 32 for C57 mice and 32 for CD1 mice except for A, E & F. n = 24 C57 mice. O. Correlation matrix of David scores across assays for C57 mice; n = 32 mice for all assays except agonistic beh [file eneuro-11-ENEURO.0342-24.2024-s008.tif]

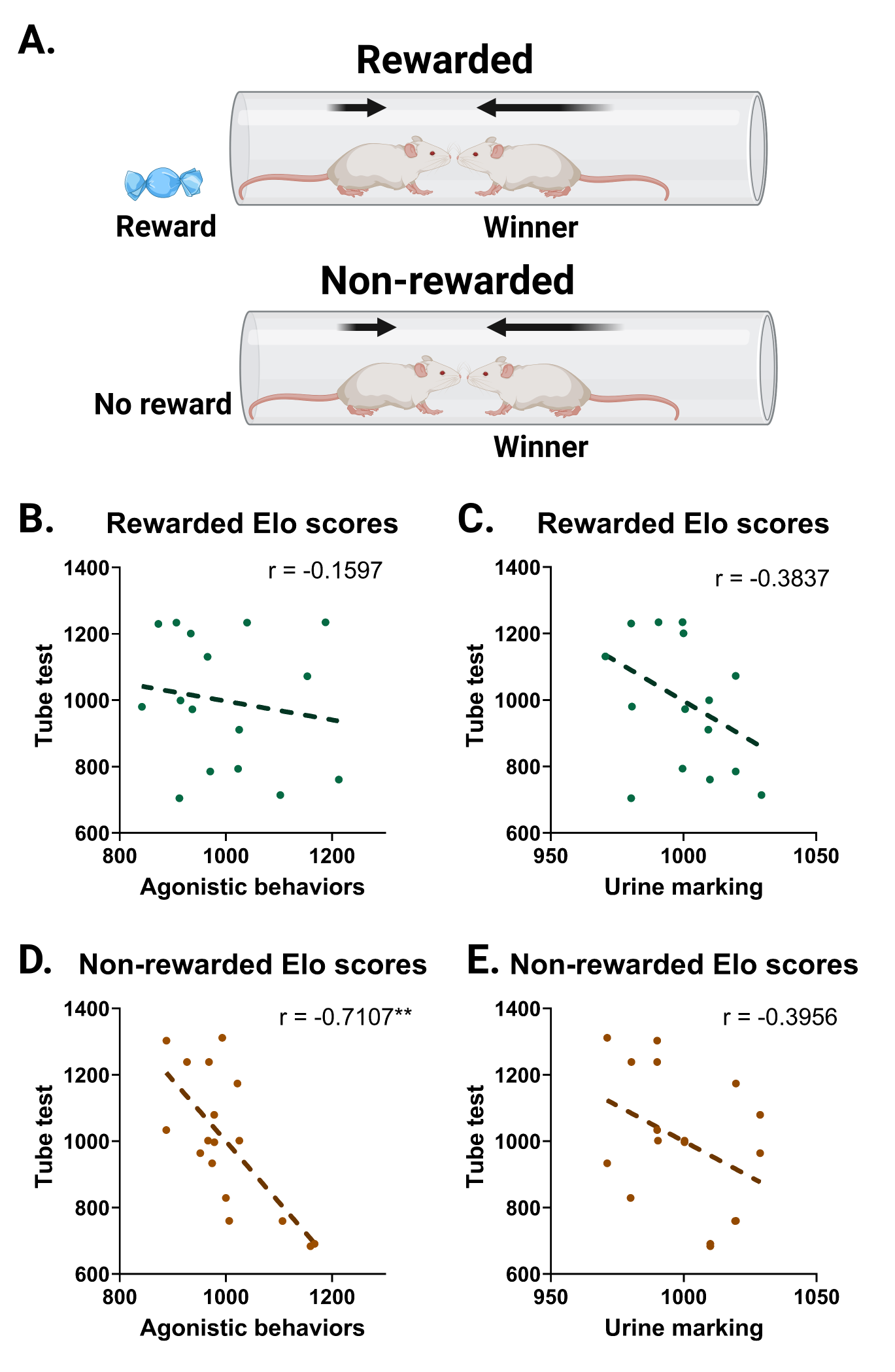

Supplement: Figure 5-3 — Tube test with reward for CD1 mice. A. Schematic of a tube test trial in which the winner is rewarded at the end of the tube for pushing the loser mouse out of the tube (rewarded) or not rewarded (non-rewarded). B-E. Elo scores across assays. For all plots, n = 16 for rewarded group and n = 17 for non-rewarded group. Linear regressions and Pearson correlations are shown. B. Pearson correlation for rewarded tube test and agonistic behavior Elo scores: p = 0.5546. C. Pearson correlation for rewarded tube test and urine marking Elo scores: p = 0.1424. D. Pearson correlation for non-rewarded tube test and agonistic behavior Elo scores: p = 0.0014. E. Pearson correlation for non-rewarded tube test and urine marking Elo scores: p = 0.1160. *p < 0.05, **p < 0.01, ***p < 0.001. Download Figure 5-3, TIF file. [file eneuro-11-ENEURO.0342-24.2024-s006.tif]
